# Achieving 9.6% efficiency in 304 nm p-AlGaN UVB LED via increasing the holes injection and light reflectance

**DOI:** 10.1038/s41598-022-04876-x

**Published:** 2022-02-16

**Authors:** M. Ajmal Khan, Noritoshi Maeda, Joosun Yun, Masafumi Jo, Yoichi Yamada, Hideki Hirayama

**Affiliations:** 1 RIKEN Cluster for Pioneering Research (CPR), 2-1 Hirosawa, Wako, Saitama 351-0198 Japan; 2 Farmroid Co., Ltd., 3-22-4 Funado, Itabashi-ku, Tokyo, 174-0041 Japan; 3 Yamaguchi University, 2-16-1 Tokiwadai, Ube, Yamaguchi 755-8611 Japan

**Keywords:** Lasers, LEDs and light sources, Optical physics

## Abstract

Crystal growth of eco-friendly, ultrawide bandgap aluminium gallium nitride (AlGaN) semiconductor-based ultraviolet-B (UVB) light-emitting diodes (LEDs) hold the potential to replace toxic mercury-based ultraviolet lamps. One of the major drawbacks in the utilisation of AlGaN-based UVB LEDs is their low efficiency of about 6.5%. The study investigates the influence of Al-graded p-type multi-quantum-barrier electron-blocking-layer (Al-grad p-MQB EBL) and Al-graded p-AlGaN hole source layer (HSL) on the generation and injection of 3D holes in the active region. Using the new UVB LED design, a significant improvement in the experimental efficiency and light output power of about 8.2% and 36 mW is noticed. This is accomplished by the transparent nature of Al-graded Mg-doped p-AlGaN HSL for 3D holes generation and p-MQB EBL structure for holes transport toward multi-quantum-wells via intra-band tunnelling. Based on both the numerical and experimental studies, the influence of sub-nanometre scale Ni film deposited underneath the 200 nm-thick Al-film p-electrode on the optical reflectance in UVB LED is investigated. A remarkable improvement in the efficiency of up to 9.6% and light output power of 40 mW, even in the absence of standard package, flip-chip, and resin-like lenses, is achieved on bare-wafer under continuous-wave operation at room temperature. The enhanced performance is attributed to the use of Al-graded p-MQB EBL coupled with softly polarised p-AlGaN HSL and the highly reflective 0.4 nm-thick Ni and 200 nm-thick Al p-electrode in the UVB LED. This research study provides a new avenue to improve the performance of high-power p-AlGaN-based UVB LEDs and other optoelectronic devices in III–V semiconductors.

## Introduction

Aluminium gallium nitride (AlGaN) based semiconductors are one of the most promising candidates for the fabrication of smart, eco-friendly ultraviolet‐B (UVB) and deep ultraviolet (DUV) emitters that would meet the requirements of the Minamata Convention of 2020^1^ and the 17 sustainable development goals (17 SDGs) of the UN^2^. Both Minamata Convention and 17 SDGs, with an aim to mitigate climate change, strive to eliminate the use of mercury vapour ultraviolet (UV) lamps in order to reduce the associated issue of CO_2_ emission^1,2^. Earlier studies show the use of UVB light of 310 nm narrow-band in cancer immunotherapy^3,4^, in vulgaris treatment^4,5^ and for plant growth with enriched phytochemicals^4,6^. Similarly, UVB light of 294 nm-band is used in the prevention of plant diseases^7,8^ and in the production of vitamin D_3_ in the human body^8,9^. Safe and smart DUV and ultraviolet-C (UVC) light sources are extremely important as a disinfectant for air, water, food and surfaces that can help to mitigate the risk of infection due to close contact with the Severe Acute Respiratory Syndrome Coronavirus 2 (SARS-CoV-2), and other similar viruses^10–15^.

Currently, toxic mercury-based UV light sources are frequently used for many industrial, agricultural and medical applications^4,8^. To replace the toxic mercury UV light sources with eco-friendly alternatives, we need to explore safe and green materials for crystal growth and device fabrication of UV light sources. Among such favourable and affordable materials, crystal growth of AlGaN compounds on AlN templates is currently being investigated for next-generation UVB light-emitting diodes (LEDs) and laser diodes (LD)^4,8,12^. Environment-friendly AlGaN-based UVB LEDs with monochromatic light emission are inevitable for both medical and agricultural applications^1–9^. The selection of green AlGaN material has many other promising features, such as high internal-quantum-efficiency (IQE) from the multi-quantum-well (MQWs) as well as reasonable level of optical and electrical properties^4,8,11,14,15^. The AlGaN-based semiconductors assist in substantial reduction of UV LED and LD production cost, partially due to their ability to be monolithically grown on low-cost c-sapphire substrates in low-pressure metal–organic vapour phase epitaxy (LP-MOVPE) reactors^4,8,11,12^. However, the direct epitaxial growth of highly conductive Si-doped n-AlGaN electron source layers (ESL), highly transparent Mg-doped p-AlGaN hole source layer (HSL) and the p-AlGaN contact-layer on the c-sapphire substrate is quite challenging. This is mainly attributed to the large lattice-mismatch of 13.3% between c-sapphire substrate and AlN epilayer as well as due to the possibility of kinetic separation while using 40–45% Al composition in AlGaN^4,8,16^.

To the best of our knowledge, limited studies have been conducted on the p-AlGaN-based UVB LEDs grown on AlN template with c-sapphire substrate^4,8,12,17–26^ which may be due to the high activation energy of Mg atoms in the p-AlGaN layer as compared with p-GaN contact layer. However, as proof of concept on III-nitride material based UVB LEDs were successfully grown on AlN template with the c-sapphire substrate, however the devices have shown quite low external-quantum-efficiency (EQE) efficiencies and maximum light output powers on a bar-wafer at RT till to date^8,17–26^, as given in Table 1. A brief report on the progress in the crystal growth and device fabrication of AlGaN/GaN-based UVB LEDs grown on AlN template with the c-sapphire substrate as studied by different research groups is summarised in Table 1^8,17–26^. The maximum EQE obtained from research conducted at the closest available UVB emission wavelength (295–300 nm) in our group is restricted to 6.5% on bare-wafer at ambient temperature^8^. On the contrary, in the UVC LEDs, about 10–20% EQE is achieved upon the usage of PSS, lenses, flip-chip (FC) process, and expensive rhodium like p-electrodes^27–29^. However, the actual maximum EQEs for DUV/UVC LEDs on bare-wafer have been reported to be around 7–8%^4,12,27–29^. A very comprehensive progress in UV light emitters and power electronic devices both theoretically and experimentally by introducing Al-graded AlGaN structures grown on c-sapphire or on SiC has been discussed elsehwre^30,31^.

**Table 1:** A comparison of the performances of AlGaN/p-GaN ultraviolet-B (UVB) light-emitting diode architectures grown on AlN template on the c-sapphire substrate, as well as their reported efficiencies and light output powers of selected next-generation UVB devices at room temperature (RT). The listed UVB light-emitting diode devices show slight variations in device design, performance, and measurement conditions.

Sources	References	UVB LED structure	Max. output power	EQE	Wavelength
CNRS	Brault et al.^17^	Sapphire/AlN/AlGaN-QD	0.25 mw @ 100 mA	0.15% @ 20 mA	305–320 nm
Kansas State University	Kim et al.^18^	Sapphire/AlN/AlGaN-QW/p-AlGaN (in-dot quick test)	1.8 mW @ 350 mA	Un-known	290 nm 305 nm
TU Berlin/FBH	Enslin et al.^19^ Rass et al.^20^ Guttmann et al.^21^ Susilo et al.^22^	Sapphire/AlN/AlGaN-QW/p-GaN (in-dot quick test)	9 mW @ 20 mA 18 mW @ 500 mA	1–2% @ 20 mA	302 nm 310 nm
Riken	Khan et al.^24^ Khan et al.^23^ Khan et al.^23^ Khan et al.^25^ Khan et al.^26^ Khan et al.^8^ Khan et al.^8^	Sapphire/AlN/AlGaN-QW/p-AlGaN (in-dot quick test)	12.5 mW @ 130 mA 7.1 mW @ 180 mA 13 mW @ 130 mA 12 mW @ 140 mA 17 mW @ 150 mA 32 mW @ 170 mA 30 mW @ 220 mA	3.3% @ 20 mA 0.5% @ 20 mA 4.4% @ 30 mA 2.4% @ 20 mA 5.6% @ 30 mA 6.5% @ 30 mA 4.7% @ 30 mA	294 nm 310 nm 295 nm 310 nm 295 nm 300 nm 310 nm
		This work	40 mW @ 170 mA	9.6% @ 10 mA	304 nm

The maximum efficiency of our previous UVB LEDs (sample-HK02) achieved so far is 6.5%. As we know that the EQE is strongly dependent on carrier-injection-efficiency (CIE), IQE in MQWs^8,32^ as well as on light-extraction-efficiency (LEE)^8,12,23,27,29^. In sample-HK02 the Al-alloy difference between QWB and quantum-well (QW) was increased from 15^26^ to 20%^8^ for better carrier confinement in the MQWs. Also, the ud-AlGaN FB layer, about 6 nm-thick, was also introduced in between the p-type MQB EBL and MQWs for better hole transport, blocking of low-energy electrons as well as for the suppression of Mg diffusion toward MQWs^8^. However, the efficiency of 6.5% in the UVB LED^8^ was limited by several key central issues such as low holes generation in p-AlGaN HSL and low holes injection toward MQWs. The low holes generation was caused by the deep acceptor level of Mg atoms in the constant Al composition level in p-AlGaN HSL and p-AlGaN contact-layer. The low hole injection was caused by the unoptimized structure of p-MQB EBL. Furthermore, the LEEs in our previous devices were restricted, due to the lack of highly transparent p-AlGaN UVB LED structure including p-MQB EBL, p-AlGaN HSL and p-AlGaN contact-layer. Further, the absorption of UVB light by Ni/Au or by thicker Ni/Al p-electrodes caused severe damages to the LEEs in the UVB devices^8,23^. We have attempted to reduce and resolve these major challenges in this work.

**Figure 1:**
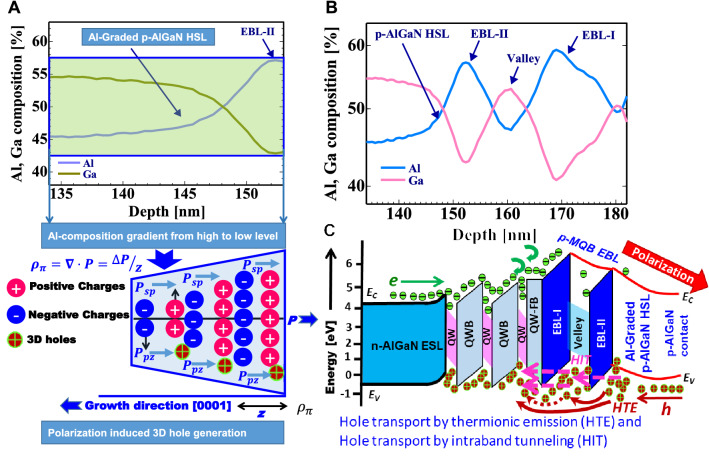
SIMS spectra of Al and Ga composition in (**a**) Al-graded p-Al_0.58_Ga_0.42_ N p-AlGaN hole source layer (HSL) (Inset: Schematic illustration of polarisation induced 3D hole generation in the Al-graded p-AlGaN HSL structure, and bandgap diagram of the graded layer, where negative polarisation charge field created by an inclination of Al-profile in Mg-doped p-AlGaN HSL), (**b**). SIMS spectra of Al and Ga composition in Mg-doped p-MQB EBL including p-AlGaN HSL, and (**c**). Estimated energy-band diagram of the newly designed p-AlGaN-based ultraviolet-B light-emitting diode (sample-COR02).

We have attempted to enhance the hole generation and transportation across the highly transparent p-AlGaN UVB LEDs. Simon et al. have reasonably improved the 3D hole generation level in the AlGaN/GaN UVC LED by polarisation effect accomplished upon distributing the Al composition profile from the p-AlGaN HSL to the p-GaN contact-layer^33,34^. Further, there is no report on better design for the highly transparent and Al-graded polarised p-AlGaN HSL for UVB emitters^4,8,23,26^. For a highly transparent and conductive p-terminal, a constant Al composition level in p-MQB EBL for Al-graded p-AlGaN HSL has been found to be unsuitable for UVB emitters^8^. Therefore, a modified design consisting of highly transparent pure Al-graded p-AlGaN HSL with a varying profile of Al composition ranging from high concentration to lower concentration is chosen as shown in Figs. 1a,b. This new design of p-side is aimed both at reducing the light absorption and to enhance the hole generation and injection toward the MQWs.

**Figure 2:**
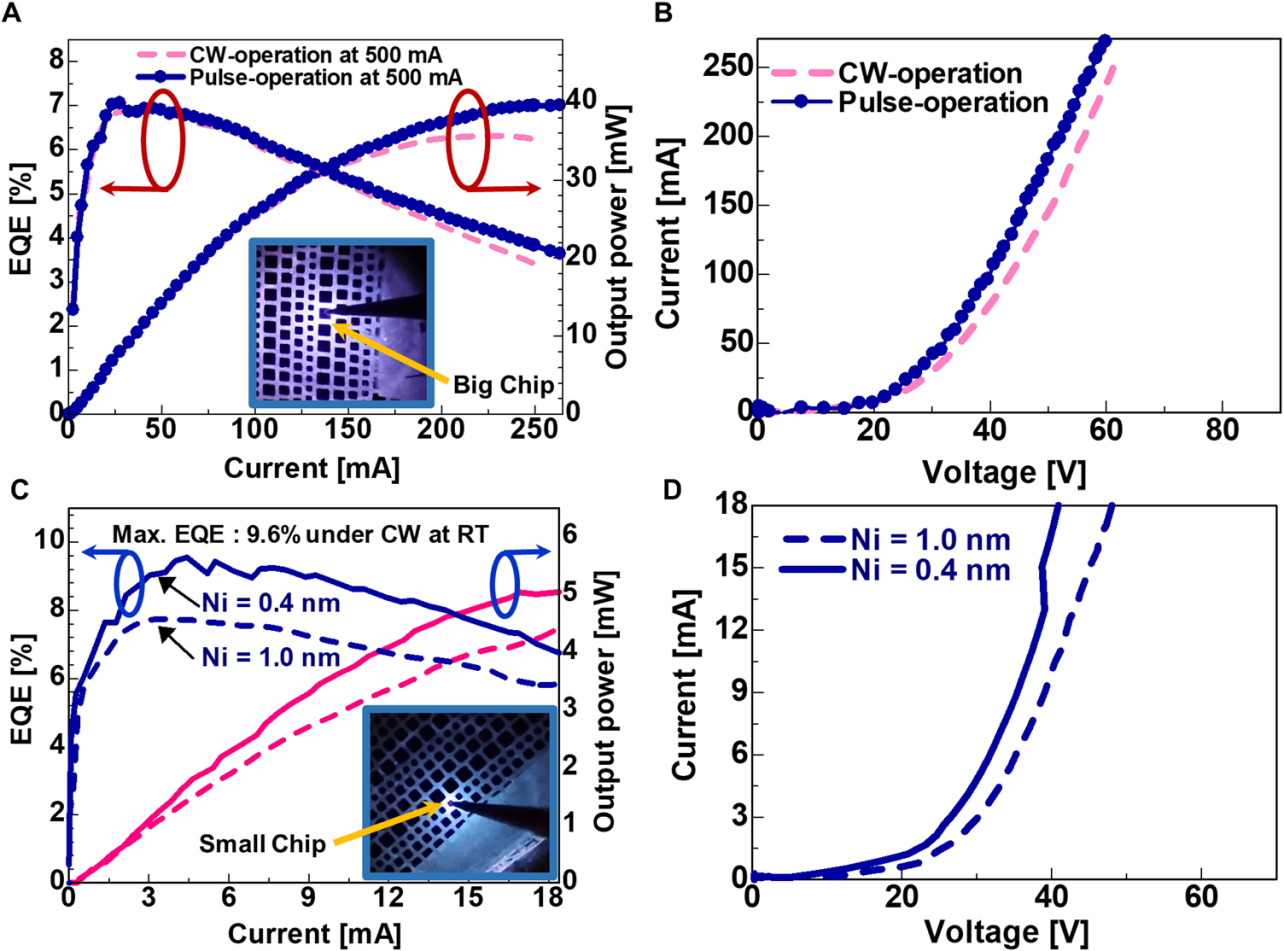
Characterisation of ultraviolet-B (UVB) light-emitting diode (LED) (sample-COR02) under continuous-wave (CW)- and pulse-operation at RT on bare-wafer with highly reflective p-electrodes: (**a**) Current vs. output power (I–L), and current vs. EQE (I–EQE) characteristics (An image of real UVB LED with big chip size: 450 × 450 μm^2^ during operation is shown in the inset), (**b**) Current vs. voltage (I-V) characteristics of UVB LED with big chip size ≈ 450 × 450 μm^2^, (**c**). I–L and I–EQE characteristics (An image of a real UVB LED with chip sizes ≈ 250 × 250 μm^2^ during operation is shown in the inset), and (**d**) I-V characteristics of UVB LED with small chip size ≈ 250 × 250 μm^2^.

Another persisting issue concerning the low reflectivity of UVB light from Ni/Au or Ni/Al p-electrode (low LEE) is also addressed in this study. From the background of optics, the maximum fraction of normally reflected UVB light from p-electrode that could be transmitted or absorbed in the p-AlGaN HSL and in the p-AlGaN contact-layer is dependent on the thickness of Ni film in (Ni/Al) p-electrode. Previously, experimental studies have been confined to 1–2 nm-thick Ni metal layer in the Ni/Al p-electrode of the UVB LED devices^8^. The validity of our approach is confirmed by comparing the computed reflectance of the UVB light with that of the experimental reflectance in the improved UVB LED in terms of measured EQEs as shown in Figs. 2a,b and 4a–d. However, theoretical modelling based on wave optics has predicted that a reduction in the thickness of Ni film in (Ni/Al) p-electrode would allow less absorption of UVB light. It would also enable achieving low contact resistance and high reflectance simultaneously for UVB emitters.

## Methods

### Low-pressure metal–organic vapour phase epitaxy

**Figure 3:**
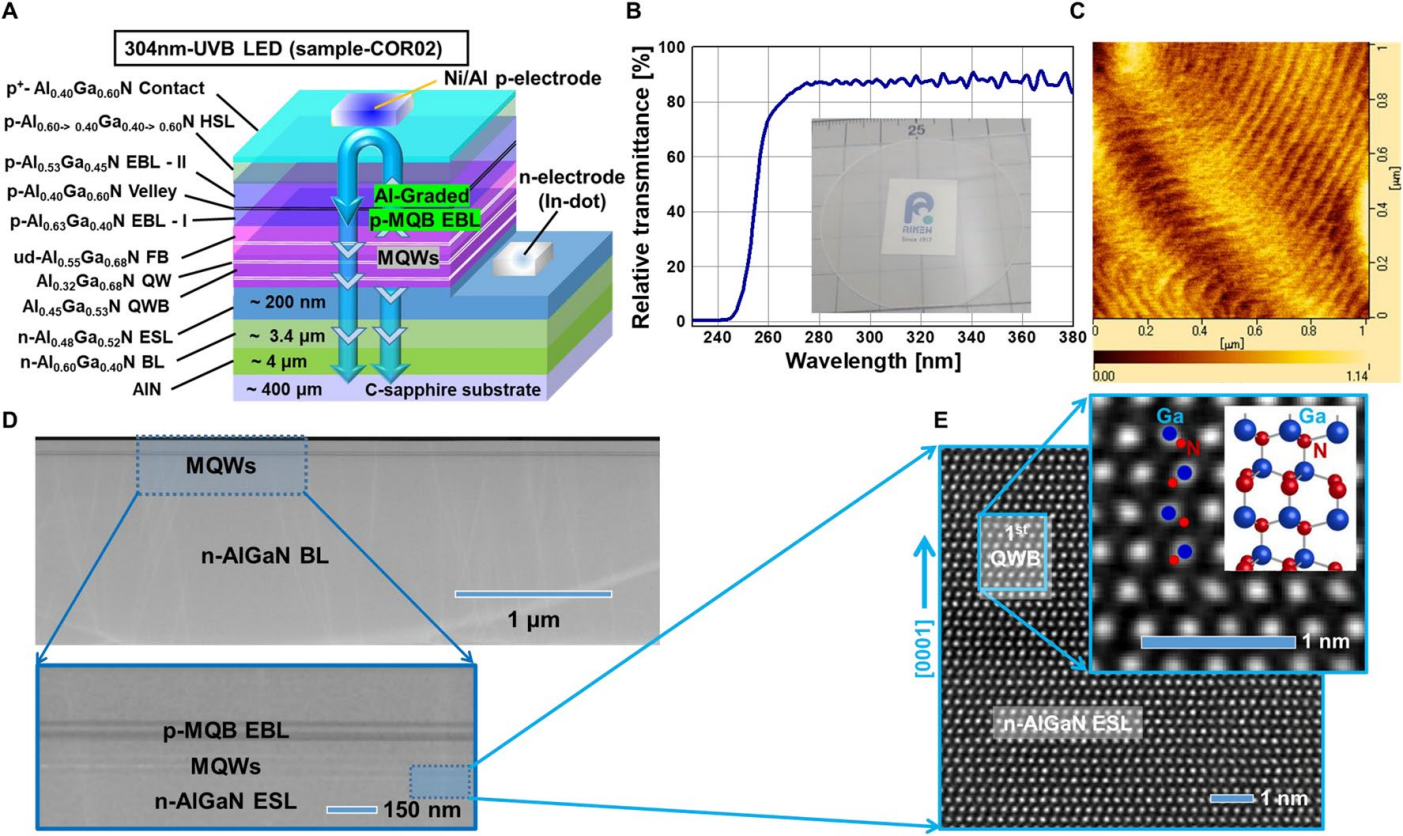
(**a**) Schematic diagram of state-of-the-art 304 nm-band AlGaN-based ultraviolet-B (UVB) light-emitting diode (LED) (sample-COR02), (**b**) Optical characterisation of the whole UVB LED (p-AlGaN-based UVB LED crystal wafer is shown in the inset), (**c**) AFM image of the final p-AlGaN contact-layer of UVB LED (sample-COR02), (**d**) HAADF-STEM image of sample-COR02, and (**e**) Magnified HAADF-STEM image of sample-COR02 taken around the n-AlGaN electron source layer (ESL) and MQWs (active region) to confirm the crystal quality at atomic resolution level.

**Figure 4:**
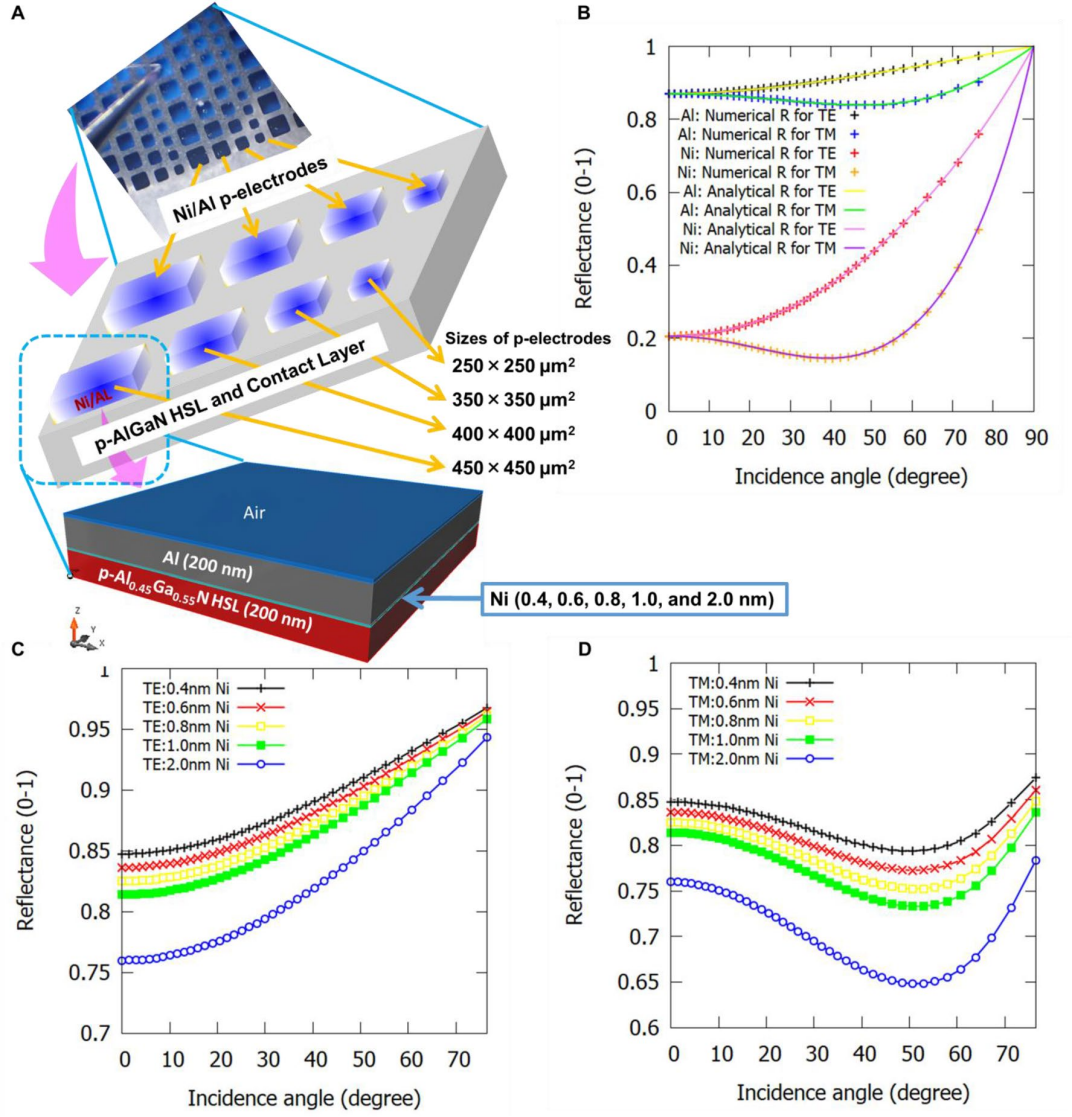
(**a**) 3D simulated unit of Ni/Al p-electrode on the top of p-AlGaN contact-layer, (**b**) Theoretical and numerical reflectance of the individual Ni and Al-metallic layer, (**c**) Incidence angle vs. reflectance from Ni/Al p-electrode as a function of varying Ni-layer thickness in transverse electric (TE)-mode, and (**d**) Incidence angle vs. reflectance from Ni/Al p-electrode as a function of varying Ni-layer thickness in transverse magnetic (TM)-mode.

In the crystal growth of improved UVB LED, Al-graded p-MQB EBL moderately doped with Mg was chosen, not only because of its utility as blocking of high-energy electrons but also to allow the hole transport via thermionic emission as well as intra-band tunnelling from p-AlGaN HSL toward the active region. The newly grown structure of Al-graded p-MQB EBL and Al-graded p-AlGaN HSL with an axially defined moderately Mg-doped p-type EBL-I (Al ≈ 63%)/valley (Al ≈ 40%)/EBL-II (Al ≈ 56%) and p-type Al-graded AlGaN HSL (Al ≈ 60–40%) in sample-COR02 as represented in Fig. 3a–e are compared with p-AlGaN UVB LEDs (sample-HK02) from the previous studies^8^. The complete schematic device structure of sample-COR02 has been shown in Fig. 3a. In previous studies, 1.7 µm-thick n-AlGaN BL underneath the MQWs of UVB LED (sample-HK02) was prepared^8^. The growth conditions of sample-HK02 are given in Supplementary Table s1. In the current study, relatively thick n-AlGaN BL (3.4 µm) in sample-COR01 (MQWs) and sample-COR2 (LED) were prepared. It is aimed to achieve high IQE in the active region after rigorous optimisation of the buffer layer. The details about the experimental growth conditions of all samples, namely, sample-HK02^8^, sample-COR01 and sample-COR2 are given, respectively in Supplementary Tables s1, s2 and s3. The first 304 nm-band UVB–MQW in sample-COR01, up to ud-AlGaN FB, was successfully grown as shown in Fig. 3a (see Supplementary Tables s2). Sample-COR01 was fabricated in an LP-MOVPE reactor by using high quality, 4 μm-thick AlN template^4,11,35^, a Si-doped n-Al_0.60_Ga_0.40_ N BL, relatively Ga-rich Si-doped n-Al_0.45_Ga_0.55_ N ESL, threefold MQWs region of Al_0.40_Ga_0.60_ N QW/ Al_0.55_Ga_0.45_ N QWB and ud-Al_0.58_Ga_0.42_ N FB as shown in Fig. 3a. Subsequently, a new design (sample-COR02) of Al-graded and moderately Mg-doped p-MQB EBL (p-AlGaN) were grown on the ud-AlGaN FB of sample-COR01. Further, in this configuration (sample-COR02), p-Al_0.14_Ga_0.86_ N-valley were sandwiched between p-Al_0.63_Ga_0.37_ N EBL-I, and p-Al_0.55_Ga_0.45_ N EBL-II to improve the holes transportation from p-AlGaN HSL toward MQWs. The Mg concentration level of approximately 2 × 10^19^ cm^−3^ was maintained in the p-MQB EBL. Finally, highly transparent, and reasonably conductive Mg-doped Al-graded p-AlGaN HSL and bulk p-Al_0.52_Ga_0.48_ N contact-layer, respectively, were grown on the overlayer of p-MQB EBL, as given in Supplementary Table s3. Mg concentration of approximately 9 × 10^19^ cm^−3^ was maintained in both the Al-graded p-AlGaN HSL and the p-AlGaN contact-layer in the sample-COR02. As a comparison, a sample-HK02^8^ that was previously grown in the horizontal LP-MOVPE reactor was utilised in the current study. The same wafer with a different chip size ≈ 350 × 350 μm^2^ of p-electrode is compared in this work (the real image of the Ni/Al p-electrodes are shown in Fig. 4a).

To enhance the reflection of UVB light from p-electrode, the thickness of the Ni underlayer in the Ni/Al p-electrode was reduced from 1 to 0.4 nm on the top p-AlGaN contact-layer of UVB LED as shown in the schematic view of Fig. 3a. For p-electrode fabrication, a low-cost conventional physical evaporation technique has been used to arrange different sizes of Ni/Al p-electrodes in a periodic array as shown in Figs. 3a, 4a. Different sizes and array pitches of the Ni/Al p-electrodes resulted in LED chips with different sizes between ≈ 250 × 250 µm^2^, 350 × 350 µm^2^, 400 × 400 µm^2^ and 450 × 450 µm^2^, respectively (the real photo of p-electrodes is shown in the inset of Fig. 4a). The high vacuum condition of approximately 4–5 × 10^−5^ Pa in the physical evaporator was successfully achieved to control the sub-nanometre-scale thickness of Ni film and also to avoid the need for Ni/Al p-electrode annealing. The details about the evaporation growth conditions of all samples are given in Supplementary Tables s4. Indium-dot based n-type electrodes were deposited on all samples to facilitate measurement on the wafer at RT, as shown in the schematic view of Fig. 3a. Indium-dot was deposited mechanically on the scribed part of n-AlGaN ESL in LED for systematic monitoring of the wafer at RT, as shown in the schematic view of Fig. 3a. The performances of 304 nm-band UVB LED devices fabricated in this study have been evaluated at RT under both continuous-wave (CW) as well as the pulse-operation using p-type electrodes with different chip sizes on the wafer as depicted in Fig. 4a. The results have been compared with the previously available sample-HK02^8^, in which uniform composition (non-graded) of Al in the p-AlGaN HSL and EBL has been used. All the reported values of light output powers in this work correspond to the total radiant flux from the UVB LEDs, which have been calibrated for the accurate performances of UVB LED devices^8,23,24^. To measure the light output power on the wafer under CW- and pulse-operation at RT, Si photodetector was set behind the test samples.

## Results and discussion

To significantly enhance the IQE, EQE and LEE in p-AlGaN UVB LEDs, a new design to improve the extent of holes injection and to increase the reflectivity by using ultra-thin Ni (0.4 nm) and Al (200 nm) p-electrode (sample-COR02) is adopted. The 3D hole generation and hole injection are increased by introducing a new Al-graded p-AlGaN HSL and Al-graded p-MQB EBL after the MQWs structure as shown in Fig. 1a,b and 3a. Several key results of the grown and fabricated sample-COR02 (Fig. 3a) are characterised and compared with our previously grown LED sample-HK02^8^. The important key design parameters that influence the LEE of the p-AlGaN UVB LEDs are also identified. In the earlier research studies, the optical transmittance (relative transmittance ≈ 90%) of UVB light through p-AlGaN HSL and p-AlGaN contact-layer with constant Al composition of AlGaN-based UVB LEDs, and the reflectance of UVB light from the p-electrode of UVB LED has been reported^8,23,26^. However, the transparency of the whole AlGaN-based UVB LEDs, including Al-graded p-MQB EBL and Al-graded p-AlGaN HSL^34^, has not been reported before. Highly transparent (relative transmittance ≈ 90%) AlGaN-based UVB LED, including Al-graded p-MQB EBL, Al-Grad p-AlGaN HSL and Al-Grad p-AlGaN contact-layer (sample–COR02), is realised for the first time which would ensure smooth transmission of the reflected UVB light (Fig. 3b). The high transparency of the various layers of AlGaN-based UVB LED is attributed to their improved crystal quality, as shown in Fig. 3d, e and better design of Al-alloy window.

Previously, Sun et al. reported the successful epitaxial growth of high crystal quality 280 nm-band AlGaN MQWs on sapphire with a misorientation angle of 4°. As a result, photoluminescence intensity was significantly enhanced for the potential applications in UVB emitters^36^. Previously, the influence of ∼ 50% relaxed n-type AlGaN ESL in 310 nm-band MQWs on PL intensity was reported^8^. However, the influence of ∼ 49% relaxed n-type AlGaN ESL in 304 nm-band MQWs on PL intensity is rarely reported in our previous work^8,25,26^. The relaxation condition in the n-AlGaN ESL underneath the MQWs, strongly influences the piezoelectricity, extended defects, point defects, Al-alloy fluctuation and nonradiative recombination centers (NRCs) in the MQWs, which ultimately degrade the IQE^4,8,16,26,37,38^. The total-TDDs in the relaxed n-AlGaN ESL (relaxation ratio ≈ 49%) has been reduced to 7 × 10^8^ cm^−2^ under the MQWs of UVB LEDs, using 3.4 µm-thick n-AlGaN BL underneath the n-AlGaN ESL (sample-COR02), as shown in Fig. 3d, e. The full width at half maxima of XRCs along the (0002) and (10–12) planes of n-AlGaN ESL (sample-COR02) have been confirmed to approximately 320 arcsec and 400 arcsec, respectively. The final p-AlGaN contact-layer surface of the sample-COR2 has been investigated by AFM studies prior to Ni/Al evaporation. The fine steps and terraces at the atomic resolution level as observed are shown in Fig. 3c. This indicates that the partially relaxed n-AlGaN ESL is quite promising in maintaining the crystalline quality until the final p-AlGaN contact-layer of the UVB LEDs is grown on the AlN template. Previous studies have rigorously evaluated the relationship between total-TDDs and the IQE in the III–V materials^39,40^. In the MQWs, the NRCs show a reciprocal relationship with IQE^39,40^. The IQE of 304 nm-band UVB–MQWs in sample-COR01 has been measured by temperature-dependent and excitation power density methods^32,41^. The maximum values of IQE in the UVB–MQWs (sample-COR01) at 100 K, 200 K and 300 K (RT) have been estimated and confirmed to be 80%, 64% and 54% (this work), respectively, as shown in Supplementary Figure V.

### Effect of Al-graded p-AlGaN hole source layer and Al-graded p-MQB electron-blocking-layer on the external-quantum-efficiency

Previously, the influence of graded Al composition on band bending in the final-quantum-barrier (FQB) of MQWs in UV LEDs was determined both theoretically and experimentally^26,42^. As a result the electron leakage was significantly suppressed, and ultimately enhanced to the performances of the UV LED devices^26,42^. Also, inverted-V-shaped quantum barriers or thinner graded quantum barriers of AlGaN-based MQWs were found to be useful for the application in DUV LEDs^43,44^. Similarly, AlGaN-based DUV LED structure with unique step-like quantum barriers in MQWs has been proposed to overcome the electron`s overshooting toward the p-side^45^. However, such experimental studies of graded Al composition in electron blocking layer or in p-AlGaN HSL of 304 nm-band UVB emitters are still rare^8,23–26^.

The accepter energy level of Mg dopant in both p-AlGaN HSL and p-AlGaN contact-layer is quite deep (≈ 450 MeV), which is caused by the low activation energy of Mg atoms in both the layers^33,34^. Owing to a large difference in the electrons and hole transport behaviour (CIE) towards the active region in III–V materials, the highly energetic electrons from the shallow donor levels can easily overshoot from the active region towards the p-region of the UVB emitters. Further, the Mg atoms can be easily diffused from the inactivated Mg atoms in p-AlGaN HSL toward the MQWs through the ud-AlGaN FB^8,26^. In UVB LEDs and LDs, the efficiency droops under high current injection and poor hole injection conditions toward the MQWs, leading to relatively poor radiative recombination^4,8^. Consequently, the performance of the UVB LEDs has been deteriorated at RT^4,8,12,23,24^, as presented in Table 1. A reasonable level of 3D hole generation has been successfully realised in the AlGaN/GaN UVC LED by polarisation effect, which is supported by the distribution of Al composition profile from p-AlGaN HSL to the p-GaN contact-layer^33,34,47^. However, both the p-GaN contact layer and Ni/Au p-electrode can absorb a significant portion of the emitted UV light^8,23^. In this regard, we have chosen a different design consisting of highly transparent Al-graded (polarised) p-AlGaN HSL as well as p-AlGaN contact-layer instead of p-GaN layers for UVB emitters. By adopting a state-of-the-art strategy of using moderately Mg-doped Al-graded p-MQB EBL in UVB LEDs, the 3D hole generation and hole transportation via intra-band tunnelling from the Al-graded p-AlGaN HSL toward the MQWs in sample-COR02 has been improved, as shown in Fig. 1.

Here, we briefly discuss the design of the Al-graded and heavy Mg-doped AlGaN HSL to realise the soft polarisation effect for the generation of 3D holes in the perspective of band engineering for 304 nm-band UVB emission. The 3D holes generation through polarisation effect shall be achieved by growing compositionally graded Al in Mg-doped p-AlGaN crystal instead of sharp heterojunction. The bound polarisation induced sheet-charge spreads to the bound 3D form by obeying the divergence law,

1 $${\rho }_{\pi }\left(z\right)=-\nabla \cdot P(z)$$

where ${\rho }_{\pi }$ is the volume charge density in the polar (z) direction, and $\nabla$ is the well-known divergence operator as depicted in Fig. 1a. When deposition is started from Ga-face crystals and graded from GaN to AlGaN, the polarisation bound charge is positive and induces the generation of mobile 3D electrons^33,34,46^ and vice versa for the generation of 3D holes. The mechanism of the generation of 3D holes by the polarisation induced phenomena is illustrated schematically in the inset of Fig. 1a. The total polarisation, constituted by the spontaneous (*P*_*sp*_) and piezoelectric (*P*_*pz*_) polarisations, can be picturised as charged dipoles in every unit cell of the crystal as depicted in Fig. 1a. Since the polarisation of Al_x_Ga_1−x_N (where x is the Al mole fraction) is higher than GaN, the sheet-charge dipoles in unit cells of the AlGaN layer are higher as compared with the GaN layer. The UVB light absorption in the devices is lesser in p-AlGaN than in p-GaN. However, the 3D holes generation in the case of transparent Al-graded p-AlGaN HSL for UVB emitters is more challenging when compared to the hybrid structure of Al-graded p-AlGaN HSL to p-GaN due to the limited availability of Al canvas variation in the former. When the composition of the layer is graded with increasing Al mole fraction, the net unbalanced bound polarisation charge is negative. It is computed from Eq. (1) to be about 5 × 10^13^ × (x_2_ − x_1_) / d cm^−3^, where x_1_ and x_2_ are the Al compositions at the ends of the graded layer of thickness d (in centimetres)^33,46,47^. Alongside the [0001]-direction (Ga-polar), the Al composition in the p-Al_x_Ga_1-x_N HSL gradually changes from high Al content of 60% down to low Al content of 40% as observed in Fig. 1a,b and the polarisation interface charge is negative. Such polarisation assisted interface negative charges induces a movable 3D hole gas as shown in the inset of Fig. 1a corresponding to sample-COR02. In the inset of Fig. 1a, the *P*_*sp*_ and *P*_*pz*_ polarisations are shown for better clarity. The Al composition of p-Al_x_Ga_1-x_N HSL of the UVB LED gradually decreases alongside the [0001]-direction (growth direction) and the 3D holes generation near the EBL-II of p-MQB EBL might be enhanced as observed from Fig. 1c. In more simple words, for the Ga-polar structure, a net negative charge exists at the Al_y_Ga_1-y_N / Al_x_Ga_1-x_N (y < x) interface, leading to the generation of 3D hole gas. These bound charges create a built-in electric field and energy-band bending that would be greater than the bandgap of the semiconductor layer if left uncompensated. To neutralise the bound, the negative polarisation charges and the holes are consequently field ionised from the neighbouring deep Mg acceptor atoms. They generate a high-density mobile 3D hole gas as shown in the inset of Fig. 1a. Ultimately, reasonably improved hole concentration of ≈ 3 × 10^16^ cm^−3^, with reduced resistivity of ≈ 22 Ω.cm and hole mobility of 9.38 cm^2^/V.s in the moderately polarised (Al: 60% > 40%) p-AlGaN HSL at RT has been achieved in sample-COR02. The specified Al composition window has been chosen to mitigate both the UVB light transmittance and the hole generation in the p-side of the newly designed UVB LED.

**Figure 5:**
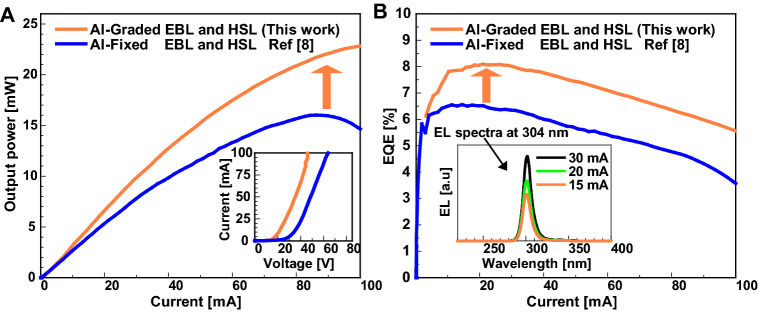
Performance evaluation of 304 nm-band ultraviolet-B (UVB) light-emitting diode (LED) (sample-COR02) and comparison with the previous UVB LED sample-HK02 (8): (**a**) I–L characteristics of improved UVB LED with big chip (*I-V* characteristics are shown in the inset), (**b**) I–EQE characteristics (single peak EL emission spectra are also shown in the inset). Some part in (**a**)–(**b**) reproduced from ref.^8^. Copyright © 2020 American Chemical Society (ACS Applied Electronic Materials).

Previously the Al composition in the EBL-I and EBL-II are maintained at the same level in p-MQB EBL of sample-HK02^8^. In order to improve further the transportation of the generated 3D holes from the p-AlGaN HSL towards the MQWs, a new design for the Al-graded p-MQB EBL structure is introduced in sample-COR02 as shown in Figs. 1b,c and 3a. In the new design, the Al composition in the EBL-I is maintained slightly higher than the EBL-II in p-MQB EBL (sample-COR02), as observed from Fig. 1b. The SIMS spectra of the Al and Ga composition in the newly designed structure of p-MQB EBL and p-AlGaN HSL is shown in Fig. 1b. The newly designed Mg-doped p-type MQB EBL would aid in the effective blocking of high-energy electron and support the holes injection through the intra-band tunnelling after the generation of 2D gas at the interfaces of EBL-I, valley and EBL-II as shown in Figs. 1b and 3a. A relatively smaller energy bandgap in the valley as compared to p-AlGaN EBL-I and p-AlGaN EBL-II in the UVB LED is chosen as visualised in Fig. 1b. In this scenario, the holes can be injected into the valley layer from the p-AlGaN HSL by both the thermionic emission and the intra-band tunnelling process and then easily be transported to MQWs^48^ as observed from Fig. 1b, c. More discussion about the transportation of holes via thermionic emission and the intra-band tunnelling process has been deeply discussed elsewhere^8,48^. In order to experimentally investigate the influence of the Al-graded p-MQB EBL and Al-graded p-AlGaN HSL on the UVB LED performance, the Ni (1 nm)/Al (200 nm) based p-electrode has been fabricated on the p-AlGaN contact-layer of the sample-COR02. The results have been compared with the previously available sample-HK02^8^, in which uniform composition of Al (non-graded case) in the p-AlGaN HSL and p-MQB EBL has been used. The performance of the sample-COR02 (this work) and sample-HK02^8^ based UVB LEDs have been measured on a bare- wafer (chip size: 350 × 350 μm^2^) at RT as shown in Fig. 5a, b. The inset of Fig. 5b shows the electroluminescence (EL) spectra of the sample–COR02 with injection drive current range from 7 to 30 mA at RT. Single peak EL operation at 304 nm emission spectra are confirmed on the wafer, where the full width at half maxima of the emitted UVB light has been controlled around 10 nm. Subsequently, the maximum light output power is remarkably enhanced from 18^8^ to 23 mW on bare-wafer (chip size ≈ 350 × 350 μm^2^) under CW-operation at RT as noticed in Fig. 5a. The inset of Fig. 5a shows the *I-V* characteristics of sample–HK02^8^ and sample-COR02 (this work) at RT. The operating voltage at 20 mA has been drastically reduced from 36 (sample-HK02)^8^ to 23 V (this work) using the same type of (Ni/Al) p-electrode. The reduction in the operating voltage is attributed to the new design of the highly conductive Al-graded p-MQB EBL and Al-graded p-AlGaN HSL of UVB LED. The maximum EQE is also significantly enhanced from 6.4%^8^ to a remarkable value of 8.2% (this work) on bare-wafer (chip size of ≈ 350 × 350 μm^2^) under CW-operation at RT, as shown in Fig. 5b. The enhancement of EQE is attributed to the suitable choice of Al composition window of 60–40% in the Al-graded p-AlGaN HSL which helps to mitigate both the UVB light transmittance and the 3D holes generation and injection in the p-side of p–n junction diode by the state-of-the-art p-MQB EBL structure as shown in Figs. 1a,c and 3a. Next, the influence of the Ni film thickness in the (Ni/Al) p-electrode on the reflectance of UVB light in LED fabricated using sample-COR02 has been investigated.

### Influence of sub-nanometre-scale Ni film’s thickness in (Ni/Al) p-electrode on light-extraction-efficiency

It has previously been established that the Al-film in (Ni/Al) p-electrode is highly reflective, but the underlying Ni film is quite problematic for the UVB light absorption and for operating voltage in (Ni/Al) p-electrode. When the Ni film of approximately 0.4 nm is introduced underneath the 200 nm-thick Al p-electrode, the performance of the UVB LEDs fabricated by using sample-COR02 is significantly enhanced. An improvement in the maximum EQE from 7.8%, corresponding to 1 nm-thick Ni film underneath the 200 nm-thick Al p-electrode, to a remarkable value of 9.6% corresponding to 0.4 nm-thick Ni film underneath the 200 nm-thick Al p-electrode on bare-wafer (chip size ≈ 250 × 250 μm^2^) under CW-operation at RT is observed as shown in Fig. 2a–d. The optical simulations which have been conducted to better understand the experimental results indicate that the strongest optical reflection of the UVB light occurs near the top of the p-AlGaN UVB LED while using 0.4 nm-thick Ni part underneath the 200 nm-thick Al of the p-electrodes as observed from Fig. 4a,d.

To precisely study the reflectance dependence of UVB light from the top (Ni/Al) p-electrode through the p-AlGaN HSL, full 3D electromagnetic optical modelling studies have been conducted as depicted in Fig. 4a,d. In the simulation model, finite-difference time-domain with the constant horizontal wavenumber approach has been used to calculate the reflectance of metallic layers in AlGaN-based UVB LEDs^49^. Figure 4a shows the schematic diagram used for the calculation of reflectance in this study. The reflectance is calculated as both the function of the thickness of Ni and the incidence angle for the transverse electric and transverse magnetic modes, by maintaining a constant value of Al-film thickness of about 200 nm as shown in Fig. 4b. For the description of the refractive indices of Al and Ni, fitting parameters based on the Lorentz–Drude model have been adopted^50^. A refractive index value of 2.58 has been used for p-Al_0.45_Ga_0.55_ N HSL crystal in this study. The unit time step, width, length and height of Yee’s unit cell, respectively, have been set as $6.419\times {10}^{-20}$ s, 1 nm, 1 nm, 0.2 nm, respectively. Modulated Gaussian waveform is selected for the source of excitation. Figure 4b shows excellent agreement in the reflectance between analytical and simulation results when light is reflected from Al-film or Ni film on p-Al_0.45_Ga_0.55_ N HSL. Figure 4c,d shows the results of reflectance in transverse electric and transverse magnetic-mode, respectively, when Al thickness is fixed around 200 nm, and only Ni film thickness is varied from 2 to 0.4 nm. The results expressed in Fig. 4c,d clearly show that a reduction in the Ni film thickness significantly increases with UVB light reflectance. With normal incidence, the Ni 0.4 nm/Al 200 nm film shows approximately 85.0% reflectance as compared to 76.0% for Ni 2 nm/Al 200 nm film. Lower Ni film thickness in Ni/Al p-electrode would ensure both low contact resistance and high reflectance in UVB emitters. More discussion on the experimental reflectance of p-electrodes is given in Supplementary note, as shown in Supplementary Figure VII.

Highly reflective layers of 1 nm thick Ni film and 0.4 nm-thin Ni film followed by a 200 nm-thick Al-film have been experimentally evaporated on the p-AlGaN contact-layer of sample-COR02 as shown in Figs. 3a and 4a and the growth conditions are given in Supplementary Table s4. Due to the high reflectivity from p-segment of the thin Ni film underneath the Al-film and due to the high relative transmittance in p-AlGaN HSL as observed from Figs. 1b and 4c,d respectively, the absorption loss in the top p-AlGaN segment is expected to be quite low. When 0.4 nm-thin Ni film underneath the Al-film of p-electrode is used in sample-COR02, the maximum light output power is remarkably enhanced to a value of 40 mW on bare-wafer (chip size ≈ 450 × 450 μm^2^) under both CW- and pulse-operation at 304 nm emission at RT as shown in Fig. 2a. Similarly, the maximum EQE has remarkably improved from 5.6^8^ to 7.2% on bare-wafer under both CW- and pulse-operation at RT, using highly reflective Ni 0.4 nm /Al 200 nm p-electrode as well as using state-of-the-art Al-graded p-MQB EBL and p- AlGaN HSL as noticed from Fig. 1a,c. The I-V characteristic of big-sized LED ≈ 450 × 450 μm^2^ under both CW- and pulse-operation has been shown in Fig. 2b. The video evidence for the performance of the UVB LED fabricated using sample-COR02 under pulse operation is provided along with supplemental material. The same phenomena of increased reflectivity by thinning of the p-segment of the Ni film under the fixed Al-film were confirmed by using optical simulation model (Fig. 4a–d) and hence the LEE was enhanced experimentally by a factor of 1.3.

Similarly, we have experimentally varied the Ni segment of the p-electrode (chip size of area ≈ 250 × 250 μm^2^) on the top of sample-COR02. After reducing the nominal p-segment thickness from 1 to 0.4 nm in sample-COR02, an approximate increase in the maximum EQE from 7.5% to a record value of 9.6% on a wafer (chip size ≈ 250 × 250 μm^2^) under CW-operation at RT was realised (Fig. 2c). Similarly, the light output power of 5 mW at 18 mA on a wafer (chip size ≈ 250 × 250 μm^2^) under CW-operation at RT has been achieved (Fig. 2c). The I-V characteristic of small-sized LED ≈ 250 × 250 μm^2^ with 1 nm-thick Ni-layer and 0.4 nm-thick Ni-layer in Ni/Al p-electrode under CW-operation has been shown in Fig. 2d. However, the operating voltages in the UVB LEDs (sample-COR2) were found to be lower with 0.4 nm-thin Ni layer when compared with the conventional 1 nm-thick Ni layer of p-electrode (Fig. 2d). The validity of our approach is confirmed by comparing the calculated reflectance with the analytical reflectance spectrum of the Ni p-electrode in the record AlGaN-based UVB LED (Fig. 4a–d) and its measured EQE (Fig. 2a,c). The values and general trends of both the simulation and experimental results agree well, although the calculated reflectance is slightly lower than the measured one throughout the spectrum. This discrepancy can be attributed to the absorption modelling and some unknown interfacial losses or internal reflections that have not been considered in the simulation. The technical maturity of the new design is validated by fabricating several working UVB LED devices on the same sample and average efficiency of 8% has been noticed. Similar results are also reproduced in separate growth experiments and processing batches of the sample. This design should be readily scalable to big wafer-sized devices and be useful for large-sized UVB LED modules for both medical and agricultural applications. Further, an EQE of more than 21% at RT is expected for the sample-COR02 by using standard LED package, PSS, resin-like lenses, photonic crystal structure, Rh-based p-electrode^27,51^ and Flip-Chip (FC) as adopted for commercial UVC LEDs^27–29^. Studies in those directions shall be pursued in the future.

In summary, pure AlGaN-based and highly transparent UVB LEDs with an efficiency of 9.6% at 304 nm spectral emission has been demonstrated on a wafer by improving the extent of holes injection and increasing the reflectivity from the ultra-thin 0.4 nm Ni and 200 nm Ni/Al p-electrode. These improvements in the efficiency of UVB LEDs are primarily attributed to the increased 3D holes generation through the Al-graded p-AlGaN HSL and holes injection through Al-graded p-MQB EBL toward the MQWs. The largely reduced dislocation density in the n-AlGaN ESL underneath the QW has caused unusual relaxation in the active region, which has mitigated the non-radiative recombination to a large extent, thereby enhancing the IQE up to 54%. The maximum EQE has drastically been improved from 6.4 to 8.2% on the wafer under CW-operation at RT. This enhancement is attributed to the Al composition window of 60–40% in the Al-graded p-AlGaN HSL incorporated by the state-of-the-art p-MQB EBL structure, which mitigates the UVB light transmittance and the 3D hole generation and transport toward the MQWs. The LEE from the highly reflective p-electrode through a transparent p-AlGaN HSL as well as p-AlGaN contact-layer are also investigated both by theoretical and experimental studies. When a 0.4 nm-thin Ni film underneath the Al-film of p-electrode (chip size ≈ 450 × 450 μm^2^) is used in the new UVB LED, the maximum light output power is remarkably enhanced to 40 mW on the wafer under both CW- and pulse-operation at RT. The maximum EQE improved from 7.2 to 9.6% on bare-wafer (chip size ≈ 250 × 250 μm^2^) under CW-operation at RT, using highly reflective 0.4 nm Ni / 200 nm Al p-electrode in combination with state-of-the-art Al-graded p-MQB EBL and p-AlGaN HSL structures. The experimentally observed improvement in the reflectivity by reducing the thickness of the Ni film-coated under the fixed Al-film matches with the predictions of the optical simulation model. The LEE has been experimentally enhanced by a factor of 1.3. High EQE of 9.6% in p-AlGaN-based UVB LEDs through careful selection of highly transparent and conductive Al-graded p-AlGaN HSL and Al-graded p-MQB EBL hold immense potential to improve the performance of UVB emitters including LDs. The research findings shall enhance the efficiency of high-power AlGaN-based UVB LEDs further by 21% when standard LED package, PSS, resin-like lenses, and FC are utilized in both medical and agricultural applications.

## Supplementary Information

Supplementary Information 1.

Supplementary Video 1.

## Data Availability

The data that support the findings of this study are available from the corresponding author upon reasonable request.

## Code availability

The numerical codes used for the numerical and theoretical reflectance are available from the corresponding author upon reasonable request.
