# Supplementary material for: Achieving 9.6% efficiency in 304 nm p-AlGaN UVB LED via increasing the holes injection and light reflectance
Source: Sci Rep. 2022 Feb 16;12:2591. doi: 10.1038/s41598-022-04876-x (PMC8850542; doi:10.1038/s41598-022-04876-x)
Supplement: Supplementary file 1 — Supplementary Information 1. [file 41598_2022_4876_MOESM1_ESM.docx]

**Achieving 9.6% Efficiency in 304 nm p-AlGaN UVB LED via Increasing the Holes Injection and Light Reflectance**

M. Ajmal Khan^1,2^*, Noritoshi Maeda^1^, Joosun Yun^1^, Masafumi Jo^1^, Yoichi Yamada^3^, and Hideki Hirayama^1^

^1^RIKEN Cluster for Pioneering Research (CPR), 2-1 Hirosawa, Wako, Saitama 351-0198, Japan

^2^Farmroid Co., Ltd., 3-22-4 Funado, Itabashi-ku, Tokyo, 174-0041, Japan

^3^Yamaguchi University, 2-16-1 Tokiwadai, Ube Yamaguchi 755-8611, Japan

^*^Corresponding author: [muhammad.khan@a.riken.jp](mailto:muhammad.khan@a.riken.jp) / khan@farmroid.com

**Supplementary Tables and Figures**

**Epitaxial Growth of MQWs and UVB LEDs**

Additional data and figures have been provided here for more information about the epitaxial growths and devices fabrications used in this work. The sample-HK02 (AlGaN-based UVB LED) used in this article for the purpose of comparative study has been taken from our previous study given in Ref^8^ and compared it with our new UVB LED (sample-COR02). In sample-HK02, a 1.7μm-thick Si-doped n-AlGaN buffer layer (BL) and then 200nm-thick Si-doped n-AlGaN electron source layer (ESL) was grown, using the crystal growth conditions given in supplementary Table s1 and the typical structure of the same device is shown in Fig. I. The Si-concentration in the n-AlGaN BL and n-AlGaN ESL layers respectively were kept at the same level of approximately 6 ×10^19^ cm^-3^. Next, three-fold multi-quantum-wells (MQWs) structure with 2 nm-thick AlGaN quantum-wells (QWs) / 8 nm-thick AlGaN-quantum well barriers (QWBs) was grown. All the QWBs of the MQWs in samples-HK02 were lightly doped with Si (TESi=0.02 sccm). Next, 6nm-thick undoped (ud)-AlGaN final barrier (FB) was grown, using the growth conditions given in Table s1. Subsequently, twofold (EBL-I and EBL-II) of p-type multi-quantum-barrier electron-blocking-layer (p-MQB EBL) between ud-AlGaN FB and p-AlGaN hole source layer (HSL) were grown. Finally, a 150 nm-thick Mg-doped p-AlGaN HSL (constant level of Al composition) including p-AlGaN contact-layer was grown (see Table s1). The Mg-concentration in the p-AlGaN HSL was kept around 6×10^19^ cm^-3^.


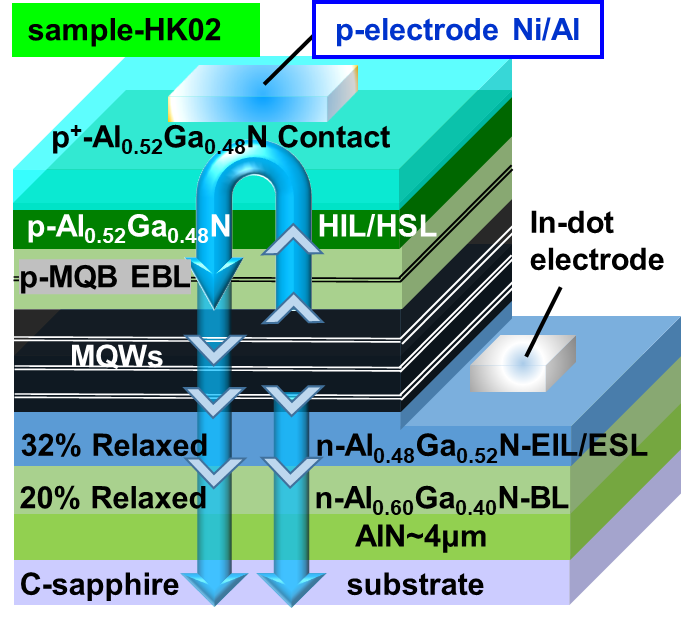


Figure. I Schematic view of 1.7 µm-thick n-AlGaN BL and 30% relaxed n-AlGaN ESL based 294nm-band UVB LEDs (location of both In-dot n-electrode and Ni/Al p-electrode is shown in the inset). Fig. I is Reproduced from ref [8]. Copyright © 2020 American Chemical Society

Table s1. Crystal Growth Conditions of the AlGaN-Based UVB LED (sample-HK02) Reproduced from ref [8]. Copyright © 2020 American Chemical Society


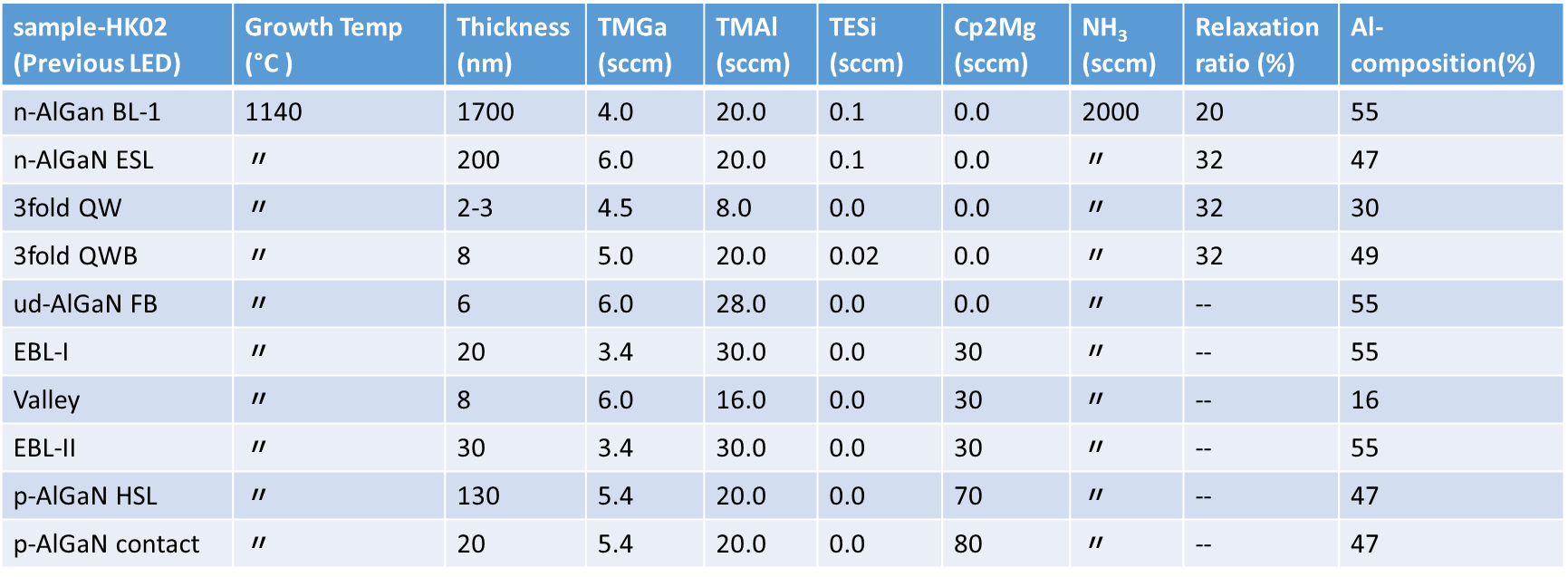


**Thickness Optimization of n-AlGaN Buffer Layer and the Influence of Strain-Relaxation on Piezoelectricity**

In sample-HK02 the relaxation condition in the n-AlGaN ESL was approximately 32% with respect to fully relaxed AlN template (Fig. II(A)). Such strained n-AlGaN ESL can influence both the crystallinity as well as piezoelectric field in the MQWs.


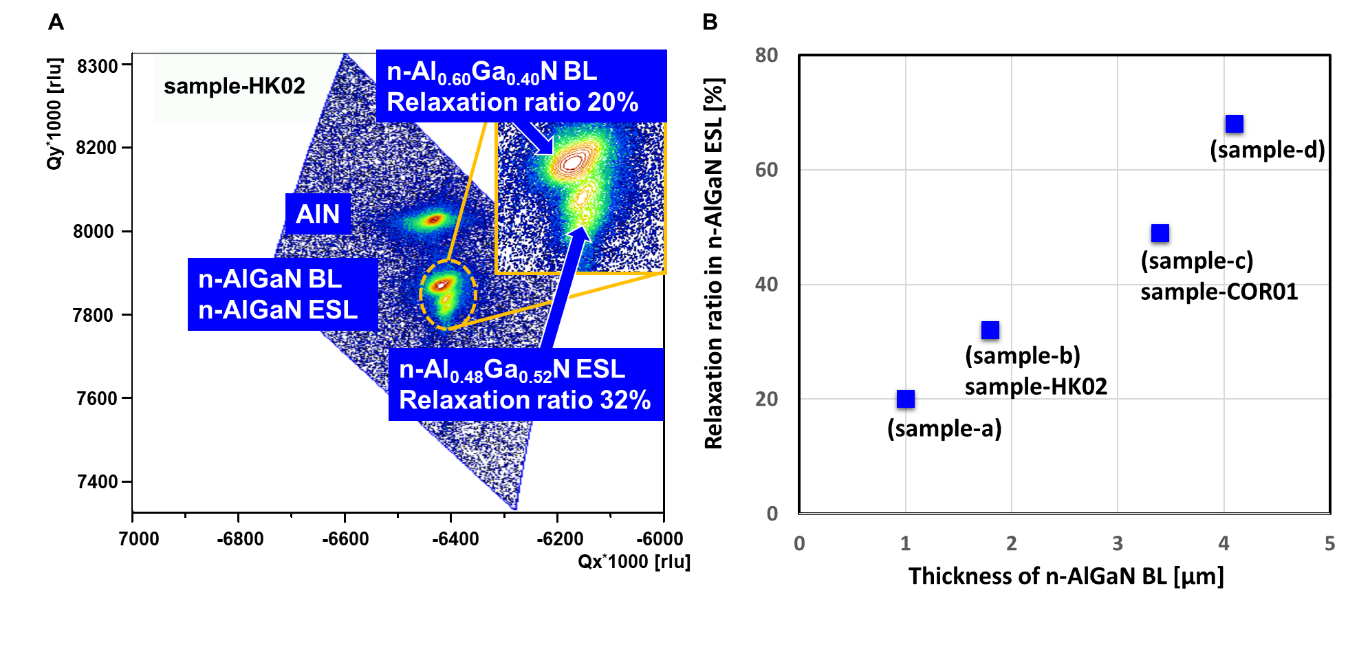


Figure. II (A) Reciprocal space mapping (RSM) along (1 1 -4) reflections of the sample-HK02, and (B) Relaxation ratio in the n-AlGaN ESL underneath the MQWs as a function of the n-AlGaN BL’s thickness at RT. Fig. II (B) is reproduced from ref [8]. Copyright © 2020 American Chemical Society

In order to avoid such highly strained conditions in the MQWs of sample-HK02, we further optimized the strain-relaxation condition in n-AlGaN ESL underneath the MQWs by varying the thickness of the n-AlGaN BL layers from 1 μm to 4 μm in the AlGaN UVB MQWs structure, as shown in Figs. II (B). We grew different samples with different n-AlGaN BL`s thickness to see the influence on relaxation condition in n-AlGaN ESL. All the four samples (n-AlGaN BLs including n-AlGaN ESL epitaxial layer) were grown with the same growth condition of MQWs in sample–COR01, except the variation of the n-AlGaN BL-1`s thickness (see Table s2). We successfully engineered the relaxation condition in n-AlGaN ESL underneath the MQWs with relaxation ratio, respectively, of approximately 20% in sample-a (1μm-thick n-AlGaN BL-1), 32% in sample-b (similar to the MQWs of sample-HK02: 1.7μm-thick n-AlGaN BL-1), 50% in sample-c (similar to the sample-COR01: 3.4μm-thick n-AlGaN BL) and 63% in sample-d (4μm-thick n-AlGaN BL-1). The reciprocal space mapping (RSM) data vs n-AlGaN BL`s thickness are shown in Fig. II (B). Hence it was confirmed that the relaxation conditions in the n-AlGaN ESL underneath the MQWs can be controlled as a function of n-AlGaN BL’s thickness, as shown in Fig. II (B). Relaxation does influence the piezoelectricity in the MQWs too. It was found that the relaxation ratio in the n-AlGaN ESL underneath the MQWs increases with an increase in thickness of the n-AlGaN BL, as shown in Fig. II(B). Such relaxation is critical for the suppression of extended defects as well as for promoting of uniform distribution of Al-alloy in the active region (MQWs) and it can have a strong effect on the desired target of emission wavelength^8^. Some theoretical relationship of the strain dependent electric field in a MQW of UV emitters^8,48^ can be derived, where the total polarization field in AlGaN layer can be expressed as:

$P=P_{pz}+P_{sp}=\left( e_{31}-\frac{c_{13}}{c_{33}}e_{33} \right)\left( \frac{a_{s}}{a_{e}}-1 \right)+P_{sp}$ (1)

where *Psp* and *Ppz* are the spontaneous and piezoelectric polarizations respectively, ae is the in-plane lattice constant of the epilayer without strain, as is the in-plane lattice constant of a virtual substrate, e31 and e33 are the piezoelectric constants, and c13 and c33 are the elastic coefficients. As we know that the strain relaxation is proportional to as (as: in-plane lattice constant), and therefore dependence of the polarization field on relaxation can be calculated by taking its derivative of Eq. (1) with respect as :

$\frac{dP}{da_{s}}=2\left( e_{31}-\frac{c_{13}}{c_{33}}e_{33} \right)\left( \frac{1}{a_{e}} \right)$ (2)

After substituting the structural information data into the Eq. (2), the relaxation dependence of the electric field was evaluated with dP/das as a function of Al-composition. Finally, it turns out that dP/das is always negative for AlGaN. Thus, the electric field in a QW was enhanced with the relaxation condition structure, which accounts for the reduced integral overlap of electron and hole wave function in the QW. Based on this physical model, it can be speculated that a partially relaxed n-AlGaN ESL up to 50% might be useful for highly efficient UVB emitters.

Next, we investigated the influence of the n-AlGaN BL`s thickness on the photoluminescence spectral intensities in UVB-MQWs. The PL spectral emission intensity were drastically enhanced with the increase of n-AlGaN BL`s thickness from 1µm to 4µm (Fig. III (B)) in sample a-d. The typical growth condition of sample-c (sample-COR01) has been provided in Table s2. Moreover, some unusual cracks in the crossed fashion

Table s2. Crystal Growth Conditions of the AlGaN-Based MQWs (sample-c = sample-COR01)


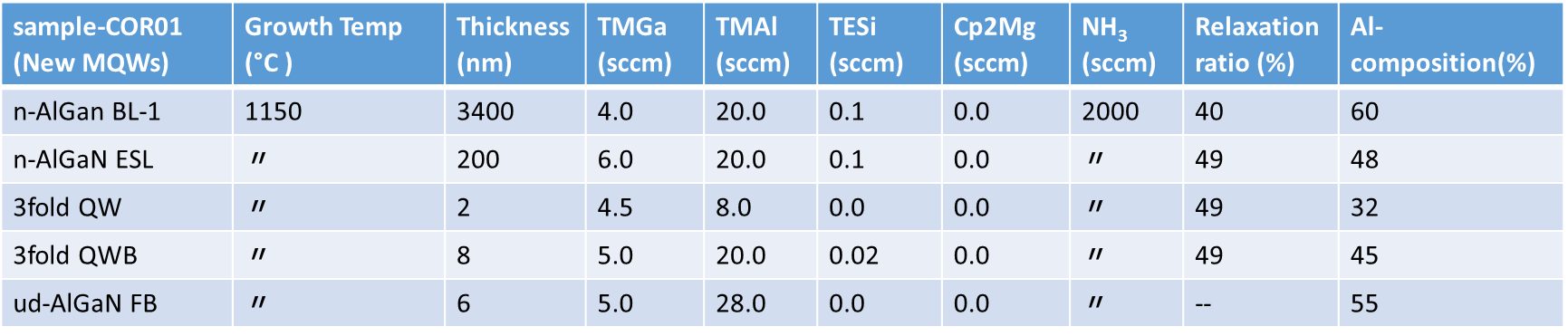


against the radial direction on the surface near to the edge area of the wafer (sample-d with 4μm-thick n-AlGaN BL-1) was observed by optical microscope (relaxation ratio approximately ~ 63%), as shown in Fig. III (D). Relatively high number of pits were also observed in the central area of the wafer of sample-d. On other hand in sample-c (sample-COR01) exceptionally low number of pits were observed, Therefore, based on the PL measurement, RSM as well as the optical microscope observation, 50% relaxed n-AlGaN ESL of sample-COR01 (3.4μm-thick n-AlGaN BL based MQWs) was found to be reasonably good choice for the crystal growth and fabrication of UVB emitters. The crystalline quality of the n-AlGaN ESL in UVB-MQWs of sample-COR01 is the best ever achieved in our lab for UVB emitters and the growth condition of the sample-COR01 are given in Table s2. As high as 54% IQE in the UVB MQWs (sample-COR01) was achieved, shown in Fig. IV(C).


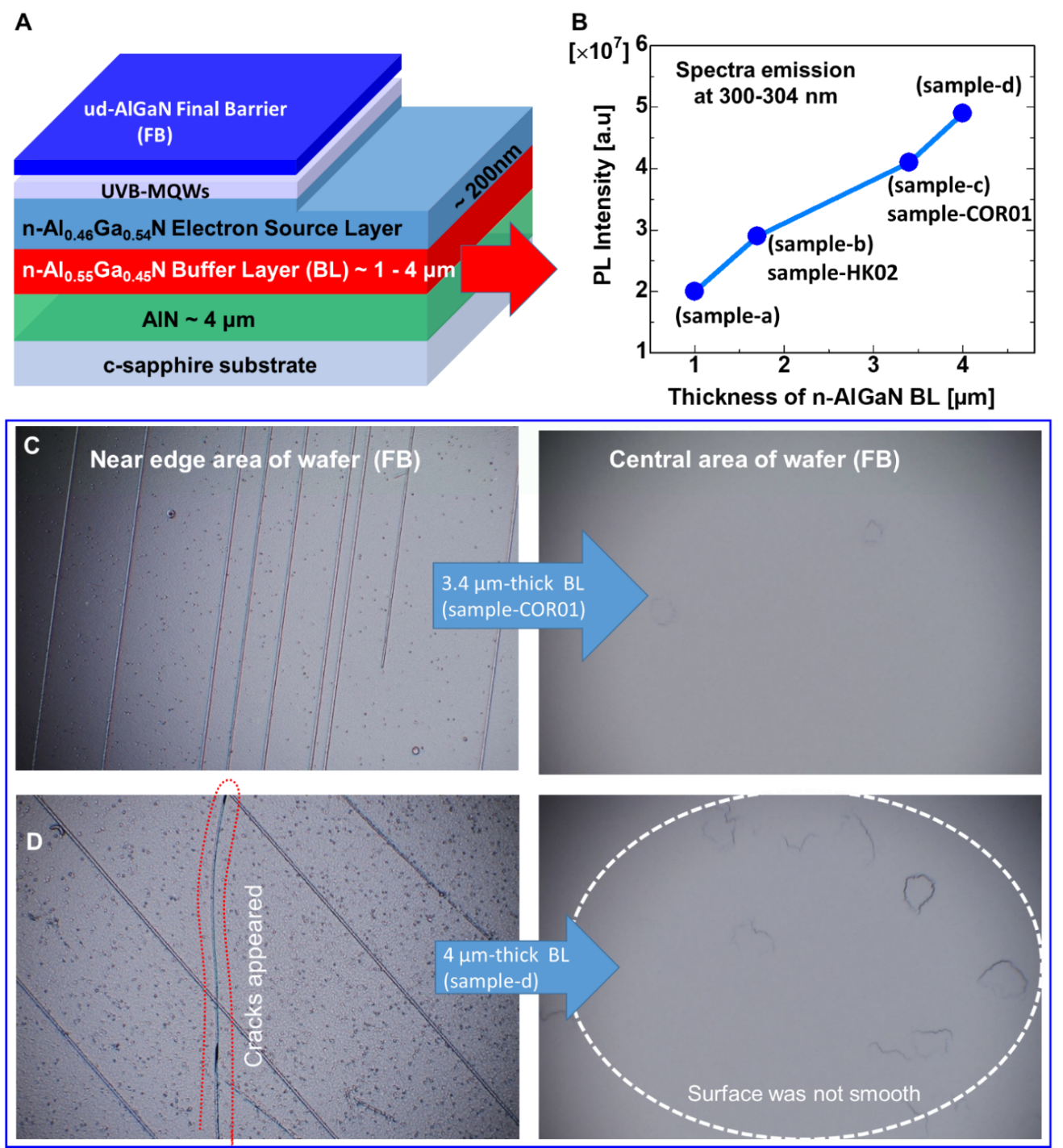


Figure. III. (A) Schematic view of (1-4 µm)-thick n-AlGaN BL and (30-61%)-relaxed n-AlGaN ESL based UVB MQWs, (B) Integrated PL spectra, respectively, of 1µm-thick n-AlGaN BL (sample-a), 1.8µm-thick n-AlGaN BL (sample-b), 3.4µm-thick n-AlGaN BL (sample-c) and 4µm-thick n-AlGaN BL (sample-d) based UVB MQWs at RT, (C). Optical microscope images around the edge and central area of the sample-COR01 (sample-c), and (D) 4µm-thick n-AlGaN BL-based MQWs (sample-d) structures grown on AlN template.

In order to understand the strain-relaxation condition in the n-AlGaN ESL, the sample-COR01 has been subjected to the reciprocal space mapping studies along (1,1,−4) reflection as shown in Figure V (A). The reciprocal space mapping image shown in Figure V(A), indicates a relaxation ratio ~ 40% in the first n-type Al_0.55_Ga_0.45_N BL and ~ 49% in the n-type Al_0.44_Ga_0.56_N ESL, respectively, which corresponds to a fully relaxed AlN substrate (a = 3.110 Å, c = 4.984 Å). In order to confirm the crystalline quality of the MQWs after partially relaxing the underneath layer of n-AlGaN ESL, the area around the interface of the first QWB of MQWs and n-AlGaN ESL has been investigated using HAADF-STEM studies on sample-COR02 as revealed in Figure V(B). The good crystalline quality of each layer of the MQWs at atomic level resolutions is confirmed in Figure V(B). The high crystal quality of MQWs with smooth interfaces is critical in reducing the NRCs and enhancing the IQE in the UVB–MQWs.


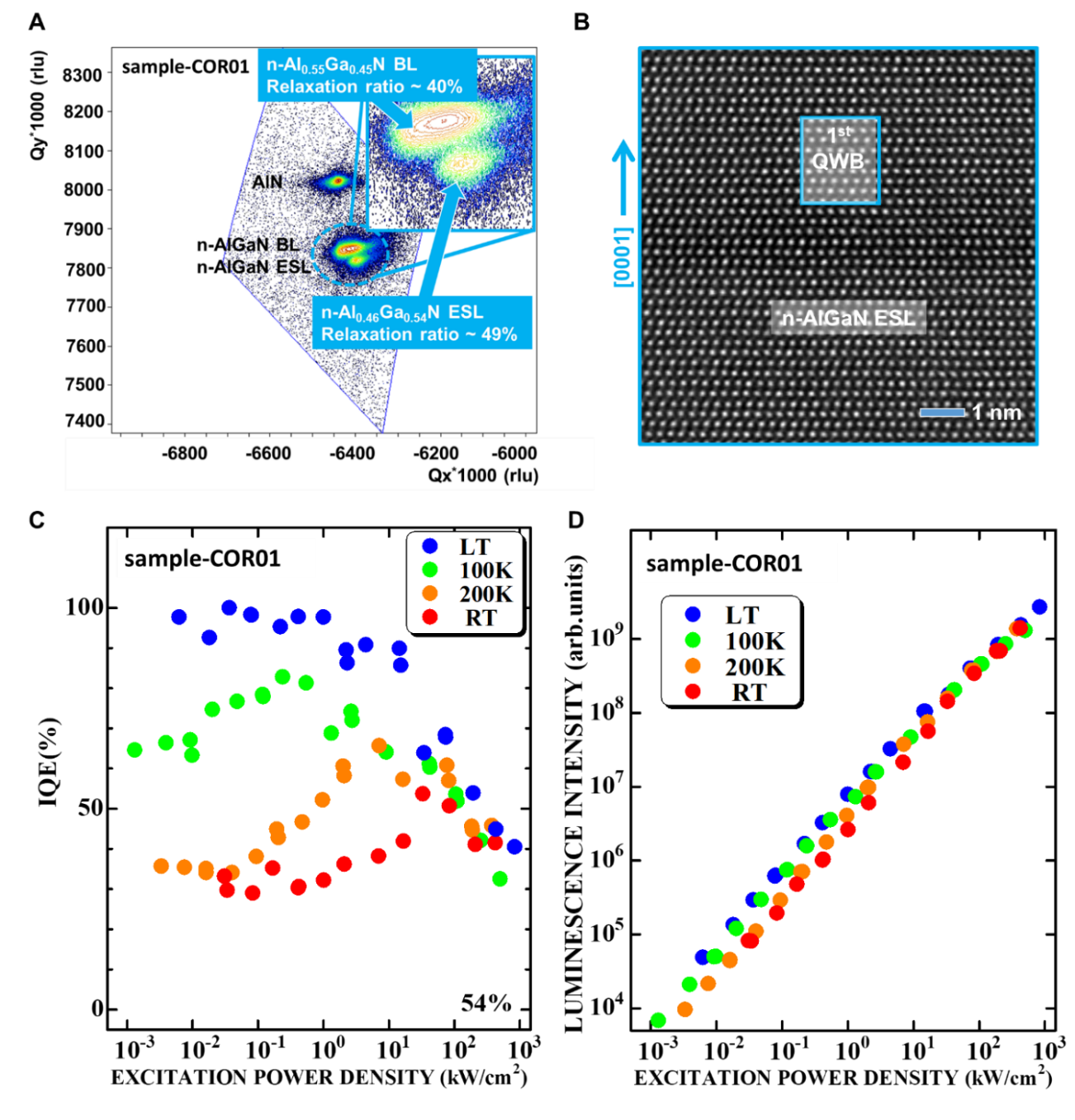


**Figure IV.** (a) A reciprocal space map along (1,1,−4) reflections of n-AlGaN BL and n-AlGaN electron source layer, where strain-relaxation state and compositional information underneath the multi-quantum-wells are shown in the inset of enlarged image (sample-COR01), (b) Magnified HAADF-STEM image of sample-COR02 taken around the interface of first quantum-well-barrier (QWB) of multi-quantum-wells and n-AlGaN electron source layer, (c) Excitation power density vs. internal-quantum-efficiencies measured at a different temperature, and (d) temperature dependence of photoluminescence as a function of the excitation power density of sample-COR01.

The IQE of 304 nm-band UVB–MQWs in sample-COR01 has been measured by temperature-dependent and excitation power density methods^32,41^. The second harmonic of a dye laser pumped by a Xe–Cl excimer laser has been used as the excitation source for the optical pumping of AlGaN-based UVB MQWs^32,41^. The integrated photoluminescence spectra at 10 K, 100 K, 200 K and 295 K (RT) for sample-COR01 as a function of excitation power density under the excitation wavelength of 240 nm have been measured and the IQEs have been estimated. The maximum values of IQE at 100 K, 200 K and 300 K (RT) have been estimated to be 80%, 64% and 54%, respectively, in the UVB–MQWs as shown in Figure V(C). The IQE curves have been normalised by the maximum IQE at 10 K and subsequently, a maximum IQE of 54% has been estimated at RT as revealed in Figure V(C). It has been noticed that the IQE remains constant at a lower excitation power density. Such observations indicate that NRCs have been in a frozen state at 10 K as observed in Figure V(C). The excitation power density dependence of the integrated PL intensity at 10 K, 100 K, 200 K and 295 K are shown in Figure V(D). At all the temperatures, the integrated PL intensity shows a positive linear relationship with the excitation power density ranging from 1 kW-cm^-2^ to 1000 kW-cm^-2^. The observations, therefore, confirm that reducing the total-TDDs^23^ in the n-AlGaN ESL from 1 × 10^9^ cm^−2^ to 7 × 10^8^ cm^−2^ results in a reasonable improvement in the IQE from 47% ^23^ to 54% as shown in Figure V(D).

Based on the relatively good crystalline quality of n-AlGaN ESL underneath the MQWs (sample-COR01), one new design of UVB LED (sample-COR02) were prepared and the growth conditions are given in Table s3. Quite high PL spectral emissions intensities in UVB LEDs (sample-COR02) were observed at RT, with band-emission wavelength of 304 nm, as shown in Figs. V(A)-(B). The first reason could possibly be due to the reduction of quantum-confined Stark effect (QCSE) in the structure with optimized QWB (8-10 nm) and QW (1-2 nm) in the MQWs and the second reason, could possibly be due to the reduction of TDDs in n-AlGaN ESL (total-TDDs ~ 7×10^8^ cm^-2^) underneath the MQWs after relaxation of 50% in n-AlGaN ESL. Furthermore, a clear shoulder peak around 270 nm was observed, which is originated from the n-Al_0.48_ Ga_0.52_N ESL, shown in Fig. V (A). Such high PL spectral emission intensity also confirm the realization of highly transparent p-AlGaN HSL including p-MQB EBL layers in p-AlGaN UVB LED.

Table s3. Crystal Growth Conditions of the AlGaN-Based UVB LED (sample-COR02)


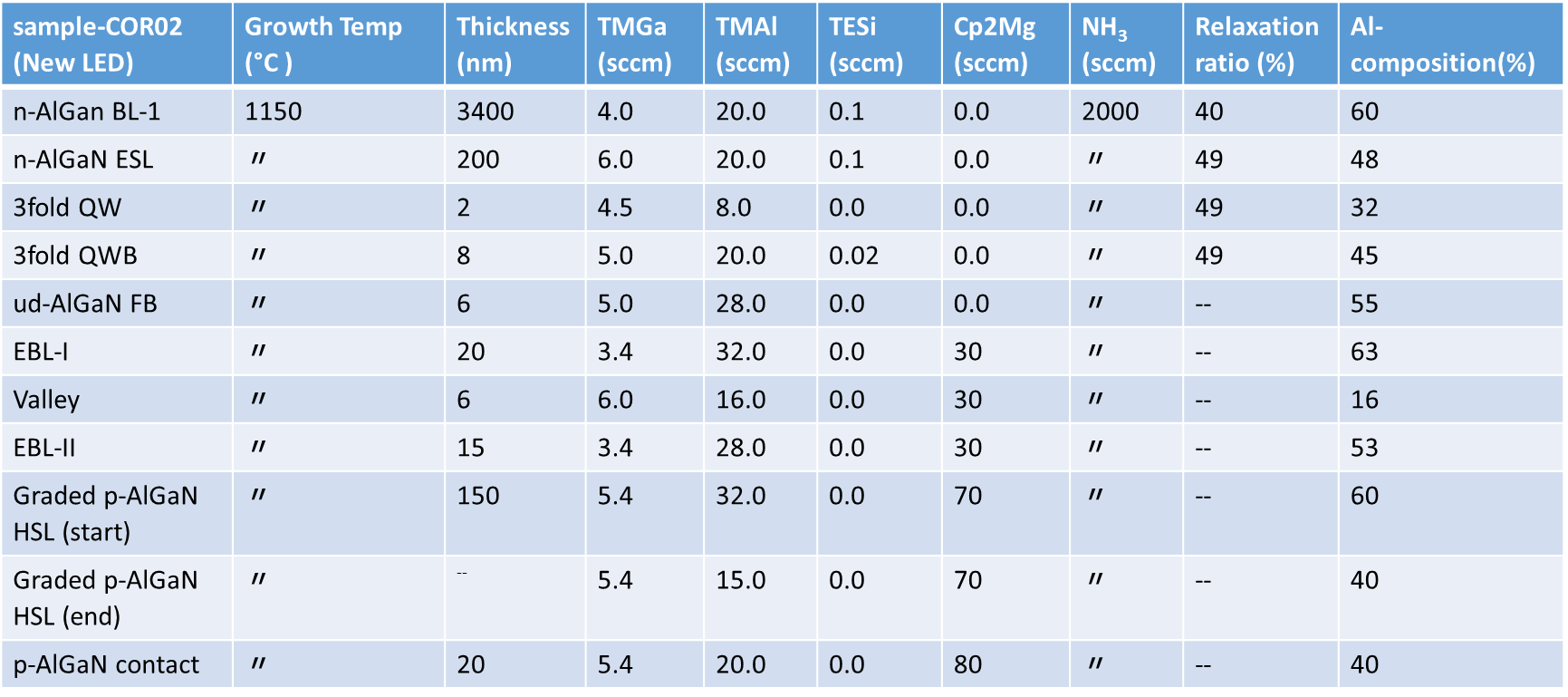


**
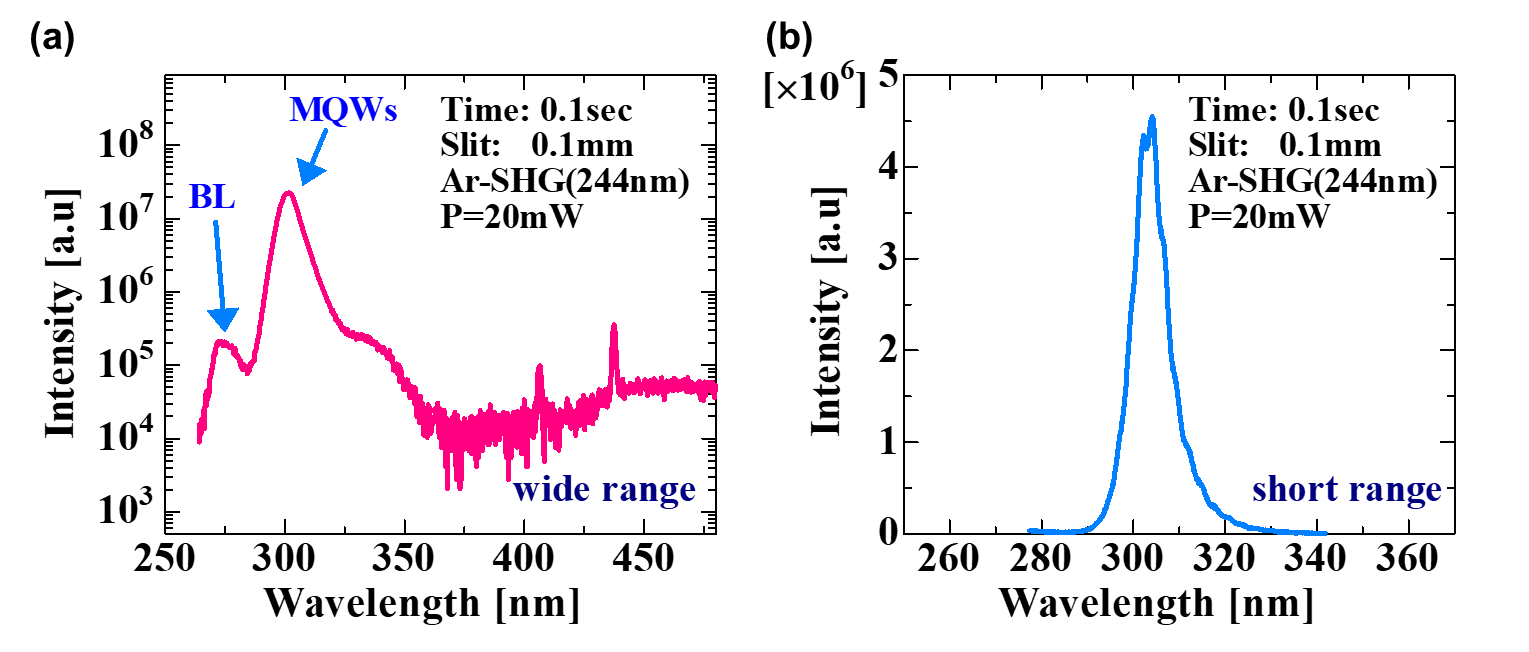
**

**A B**

Figure. V Integrated PL spectra of sample-COR02, (a) wide range in log-scale and (b) short range in linear-scale, measured using a 20mW Ar-SHG (244 nm) laser as an excitation source at room temperature (300K).

transmittance and the hole generation in the p-side of the newly designed UVB LED.

In order to improve the transportation of the generated 3D holes from the p-AlGaN HSL towards the MQWs, a new design for the Al-graded p-MQB EBL structure is introduced in sample-COR02 as shown in the main article of Figures 1b, 1c. The SIMS spectra of the Al and Ga composition in the newly designed structure of p-MQB EBL and p-AlGaN HSL is shown in the main article of Figure 1b. The Al composition in the EBL-I is maintained slightly higher than the EBL-II in p-MQB EBL, as observed from Figure 1b. The newly designed Mg-doped p-type MQB EBL would aid in the effective blocking of high-energy electron and support the holes injection through the intra-band tunnelling after the generation of 2D gas at the interfaces of EBL-I, valley and EBL-II as shown in Figure 1b. A relatively smaller energy bandgap in the valley as compared to p-AlGaN EBL-I and p-AlGaN EBL-II in the UVB LED is chosen as visualised in the main article of Figure 1b. The major hole transport in the conventional bulk p-AlGaN EBL in the UVB LED occurs predominantly through the thermionic emission. This is strongly dependent on the valence band offset and the hole concentration, given by the relationship,

$\boldsymbol{\emptyset}_{h}=\Delta E_{V}-kT*ln(p/N_{v})$ (3)

where, $\emptyset_{h}$ is the valence band barrier height, $\Delta E_{V}$ is the valence band offset,$k$ is the Boltzmann constant, $T$ is the carrier temperature, $p$ is the hole concentration and $N_{V}$is the effective density of states for holes. The thin p-AlGaN valley occurs with a smaller energy bandgap between p-AlGaN EBL-I and p-AlGaN EBL-II as noticed in the main article of Figures 1b, 1c. When the valley is precisely inserted near the softly polarised Al-graded p-AlGaN HSL, the holes concentration can be increased into the p-MQB EBL which in turn decreases $\emptyset_{h}$ as observed in the main article of Figures 1a, 1b. In this scenario, the holes can be injected into the valley layer from the p-AlGaN HSL by both the thermionic emission and the intra-band tunnelling process and then easily be transported to MQWs as observed in the main article of Figures 1b, 1c. Alternatively, the polarisation fields have been exploited to create a parallel sheet of 2D hole gases in the Mg-doped p-MQB EBL, as it has been created in the AlGaN/GaN multiple-QW structures in 1998^48^. The polarisation field effect, which is generated at the interfaces of the p-AlGaN EBL-I (QB), p-AlGaN EBL-II (QB) and valley (between Valley and QB) can be expressed as^48^,

$\Delta P=P^{QB}-P^{Valley}$ (4)

Due to the polarisation field effect, a high $p$ value can be obtained in the valley, which helps in decreasing the valence band barrier height$\emptyset_{h}$, for holes injection from the p-AlGaN HSL toward the MQWs.

**Evaporation of Highly reflective p-electrodes on p-AlGaN contact-layer**

We used a cleaned conventional physical vacuum evaporation system for metal evaporation with relatively good background pressure of 4-5 × 10^-5^ Pa, which is equipped with standard INFICON SQM-160 Rate/Thickness monitor system, shown in Figure. VI. The SQM-160 uses proven INFICON quartz crystal sensor technology to measure rate and thickness in thin film deposition processes. Two sensor inputs are standard and four additional sensor inputs are optional. Growth rate displays of 0.1Å/s or 0.01Å/s are user selectable and possible. In INFICON, four relay outputs allow the SQM-160 to control source or sensor shutters, signal time and thickness set points, and signal crystal failure. Digital inputs allow external signals to start/stop and zero readings. Based on this mechanism we optimized the growth condition of Ni/Au, Ni/Mg, Ni/Al and Rh in our lab at Riken, shown in Figure. VI.

In this paper, we showed the results of reflectance, when Al thickness is fixed around 200 nm, and only Ni film thickness is varied from 2 nm to 0.4 nm. It was discovered during the growth and characterization of Ni/Al based UVB LED that more thinner beyond 0.4 nm is not suitable because of the surface roughness of p-AlGaN contact-layer prior to evaporation of Ni-layer. We carefully controlled and monitor to the thickness of Ni and Al-layers in the p-electrodes used in this work, as given in Table s4. Our physical vacuum evaporation system is equipped with several boats for ranging from Ni, Au, Al, Ti, Rh and Mg respectively, as shown in Figure. VI.


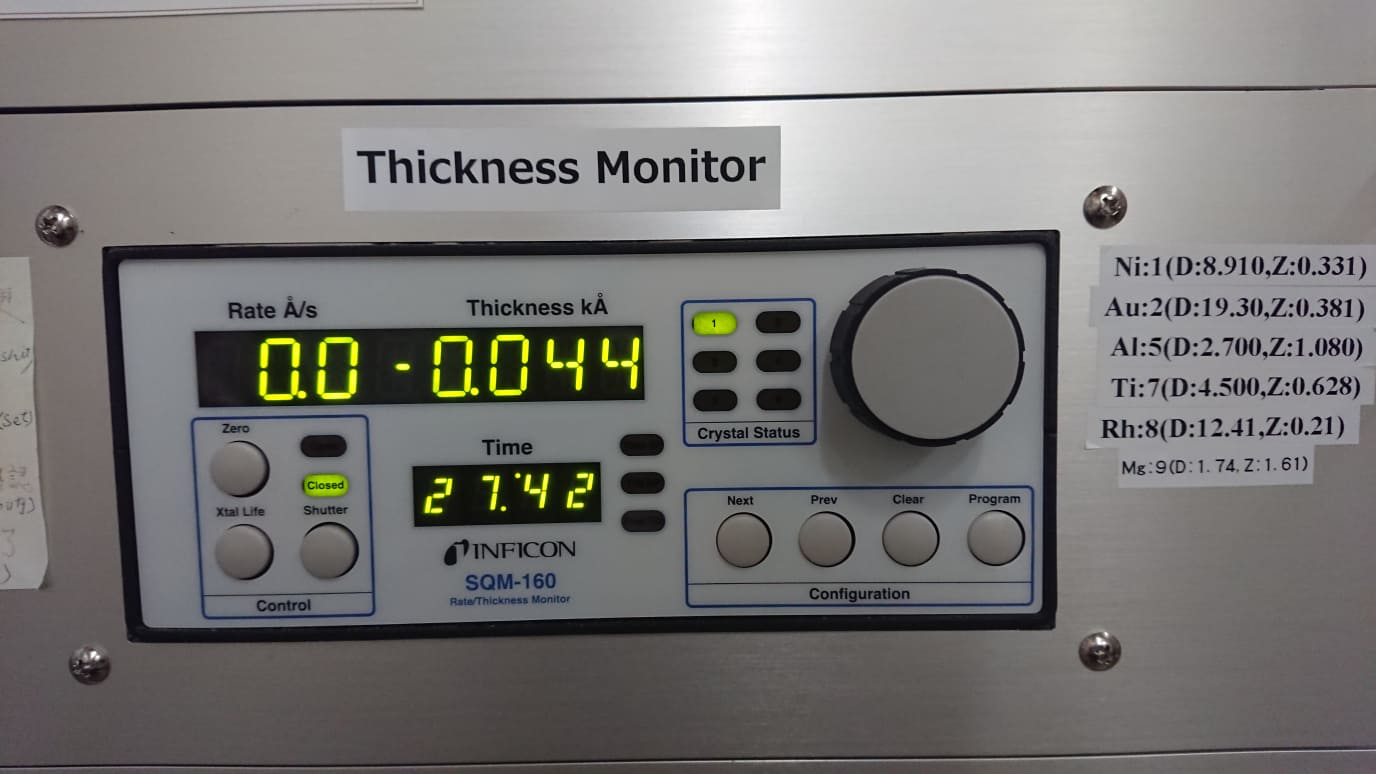


**Figure. VI** The SQM-160 uses proven INFICON quartz crystal sensor technology to measure rate and thickness.

In this work, different thickness (2nm->0.4nm) of Ni-layer in the Ni/Al p-electrode were carefully controlled using INFICON quartz crystal sensor technology to measure rate and thickness, as given in Table s4.

We achieved quite smooth surface of p-AlGaN contact-layer in highly efficient LED sample-COR2, prior to the evaporation of Ni/Al p-electrodes, as shown in the AFM image of Figure. 3c given in the main body of the research paper. It is worthy to mention here that evaporating thickness of Ni layer < 0.4 nm in p-electrode on p-AlGaN contact-layer was not suitable. Because, we were unable to get high efficiency in sample-COR02 due the surface roughness limitation of p-AlGaN contact-layer as well as due to the issue of Al-layer peeling off in Ni/Al p-electrodes.

Table s4. Different Growth Conditions for different thickness of Ni/Al p-electrodes.


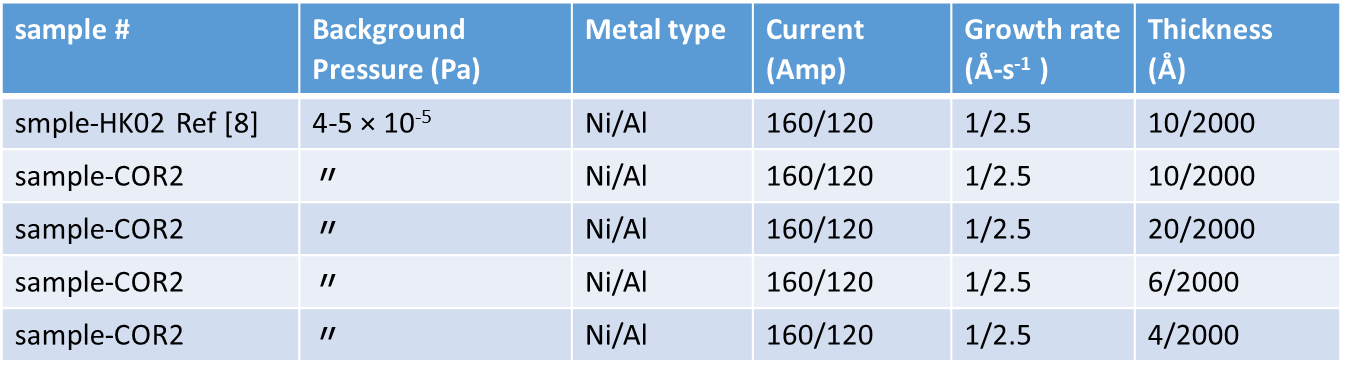


The Ni-layer thickness beyond 2 nm was found to be unsuitable too for UV emitters, and it was discovered by Maeda et al. that due to the absorption of UV light in the thicker Ni-layer in DVC LED^51^ the LEE was reduced. Pervasively, the individual reflectance of each metallic layers were measured as shown in Figure VII (A), where the schematic diagram of the samples were used during the investigation and evaluation of the reflectivity from Ni/Mg, Mg, Ni/Al and Al p-electrodes were also given. Next, different p-type electrodes like Ni(1nm)/Mg(200nm), Mg(200nm), Ni(1nm)/Al(200nm), and Al(200nm) were evaporated on the sapphire substrates^23^. Subsequently the reflectivity from Ni/Mg, Mg, Ni/Al and Al p-electrodes were measured experimentally by using standard optical measurement system^23^. Figures VII (a)-(b) shows the relative reflectance of Ni(1nm)/Mg(200nm), Mg(200nm), Ni(1nm)/Al(200nm), and Ni(30 nm)/Au(150nm) layers directly deposited on the c-sapphire substrates and calibrated by the reflectance of an Al/sapphire reference sample^23^. The experimental measured values of the relative reflectivity for UVB emission around 300 nm are approximately 1.03, 0.88, 0.83, and 0.34 for the Mg, Ni/Mg, Ni/Al, and Ni/Au electrodes, respectively, as shown in Fig. VII (B) and could be higher for the upper bound emission of 315 nm in UVB LED devices. However, we found some oxidation issue with Ni/Mg p-electrode and it was discarded from UVB LEDs.

**A B**


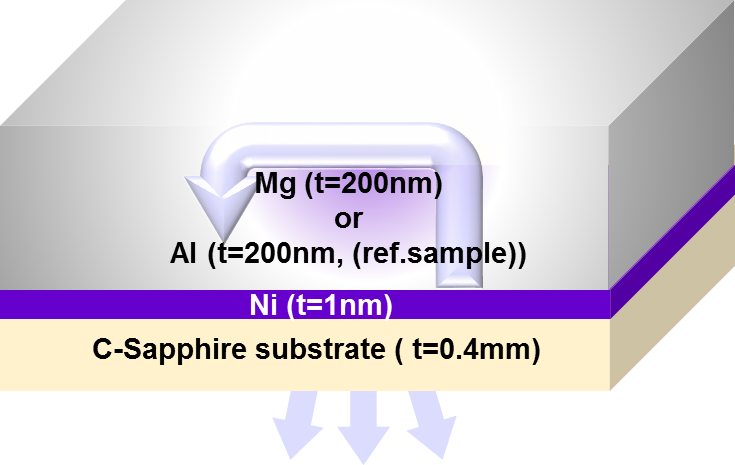

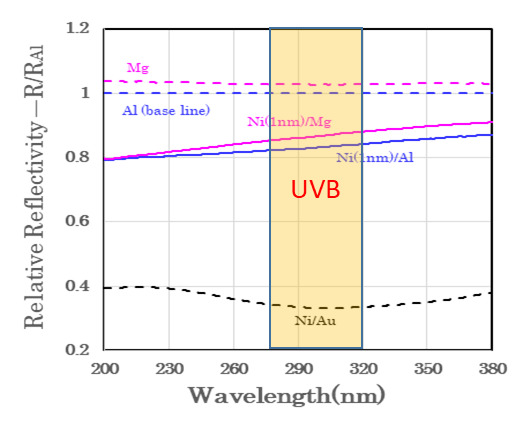


Figure. VII Schematic diagrams of the test samples of p-electrodes used for evaluating the reflectivity from (a) Ni/Mg, Mg, Ni/Al and Al, layers directly deposited on sapphire wafers, and (b) relative reflectance of Ni(1nm)/Mg(200nm), Mg(200nm), Ni(1nm)/Al(200nm), and Ni(30nm)/Au(150nm) stacking layers directly deposited on sapphire substrates calibrated by the reflectance of an Al/sapphire reference sample. **Figures: VII (a)-(b) reproduced from [23]. © 2019 The Royal Society of Chemistry (RSC).**

Previously, Maeda et al.^51^ also investigated to the increases the LEE of 280 nm band DUV LEDs by introducing highly reflective Ni/Mg and Rh p-type electrodes. The EQEs of the LEDs were increased by factors of about 1.6 and 1.4, respectively, by replacing the conventional Ni/Au p-type electrodes with highly reflective Ni/Mg and Rh electrodes. Rh as p-electrode showed quite promising results, but it is still under investigation for reproducibility and reliability purposes, because the evaporation of Rh p-electrode is quite difficult in the conventional evaporation system due its high melting point around 1964 °C.
